# Supplementary material for: Identification of four novel associations for B-cell acute lymphoblastic leukaemia risk
Source: Nat Commun. 2019 Nov 25;10:5348. doi: 10.1038/s41467-019-13069-6 (PMC6877561; doi:10.1038/s41467-019-13069-6)
Supplement: Supplementary file 2 — Supplementary Information [file 41467_2019_13069_MOESM2_ESM.pdf]

Identification of four novel associations for B-cell acute lymphoblastic leukaemia risk

Vijayakrishnan *et al.*

Supplementary Information

Supplementary Figure 1: Overall study scheme.

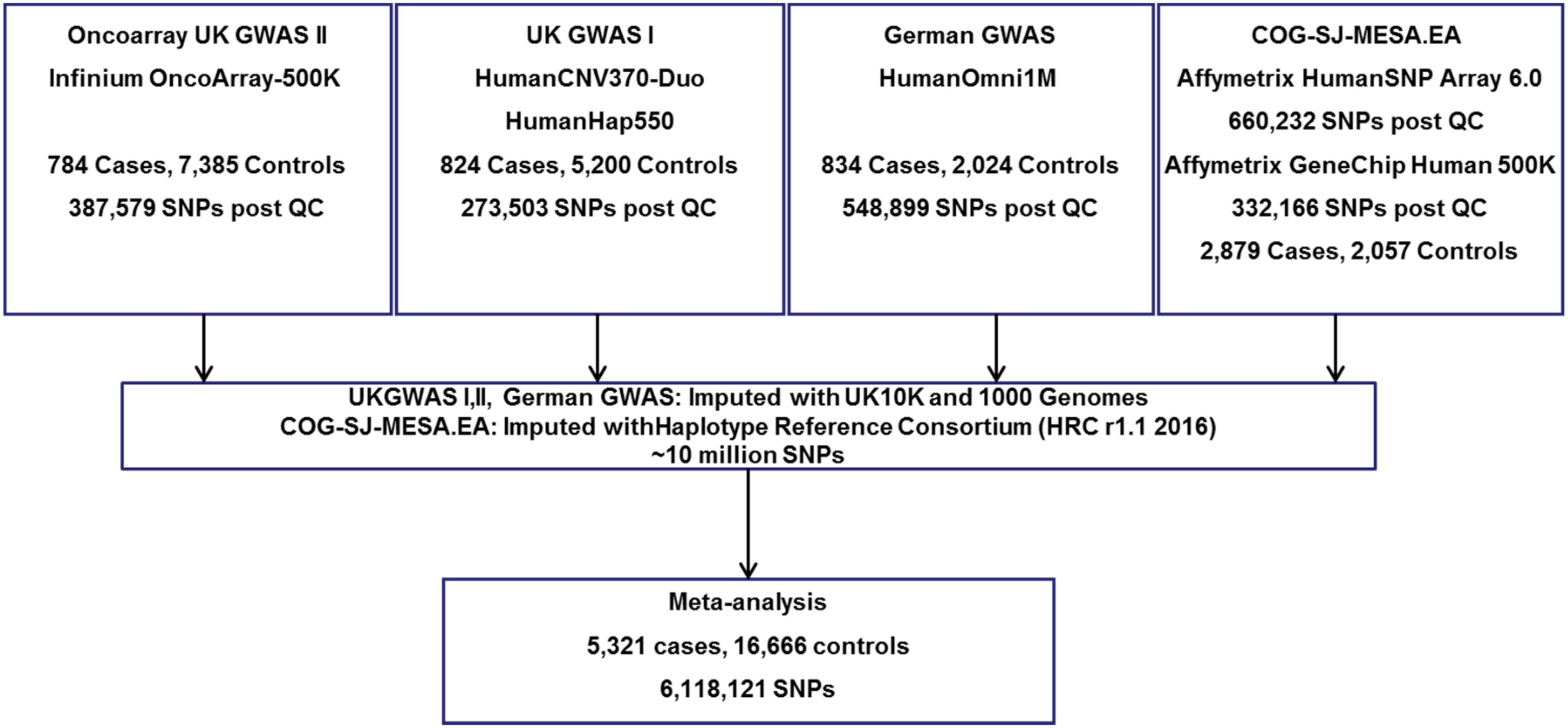

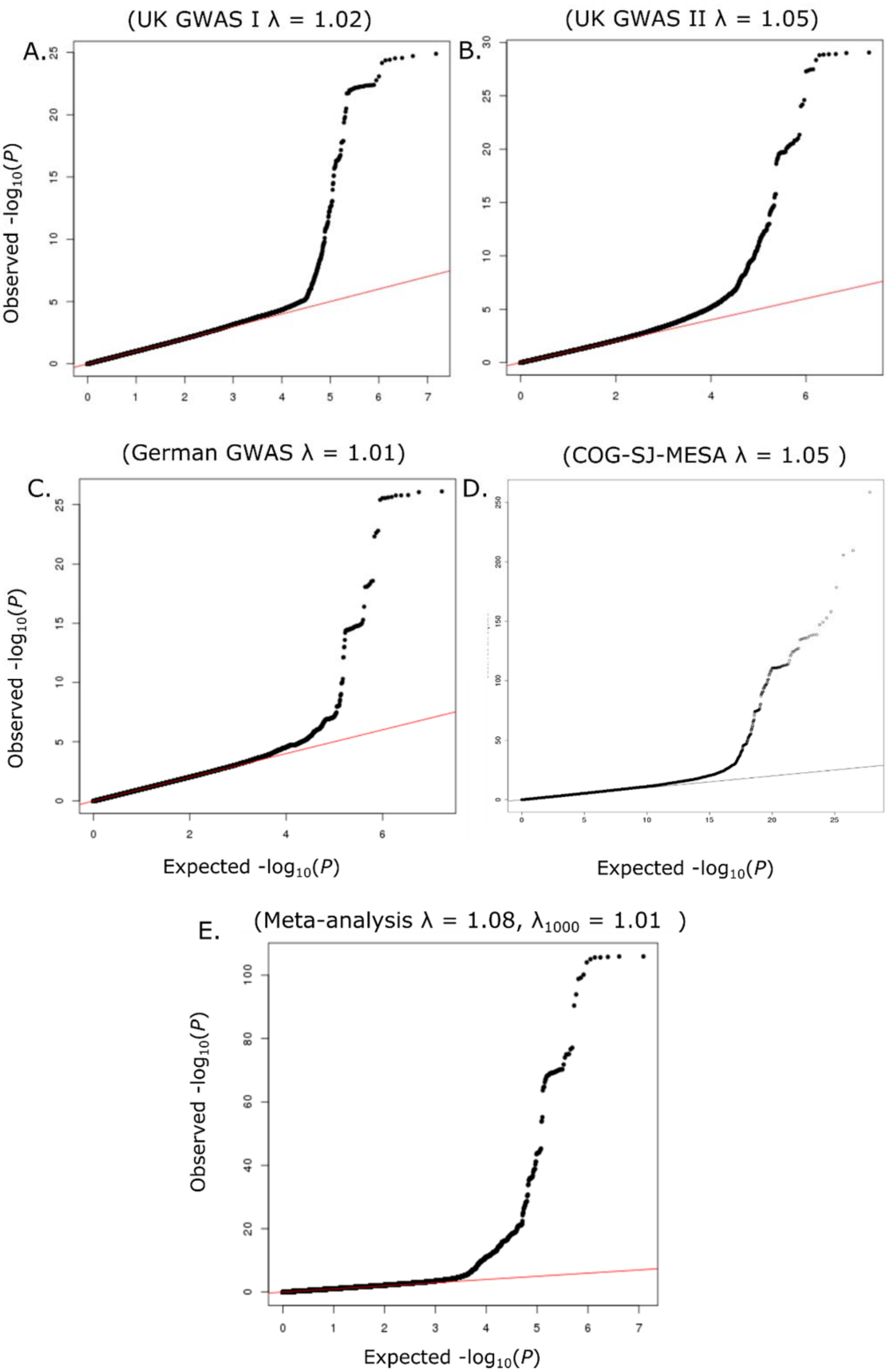

Supplementary Figrue 2 : QQ plot post imputation and genomic corrections for (A) UK GWAS I, (B) German GWAS, (C) UK GWAS II, (D)COG-SJ-MESA and (E) Meta-analysis showing ʎ values.

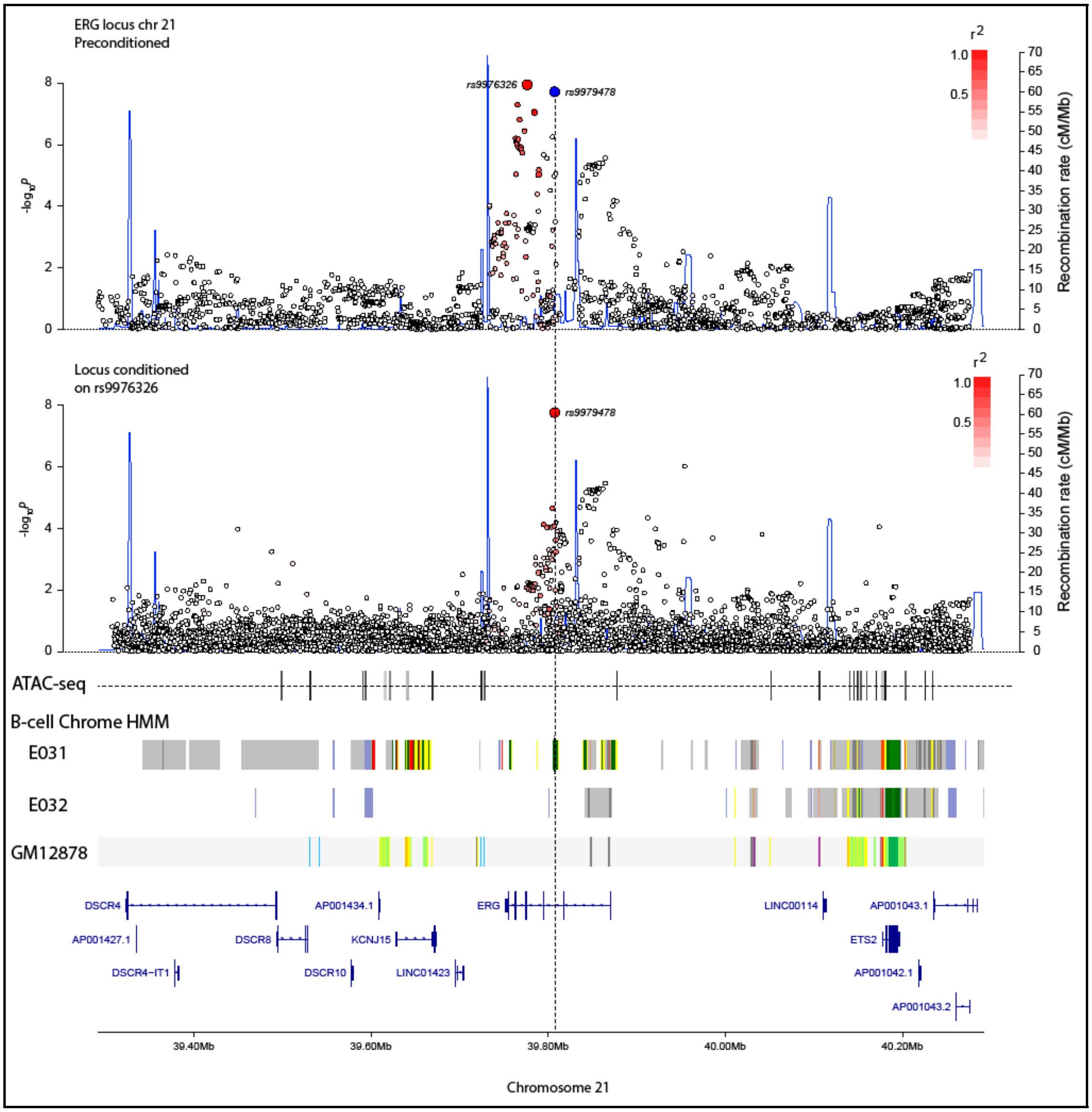

Supplementary Figrue 3: Pre and post conditional analysis association plots with epigenetic annoation. Top pane shows the chr21q22 locus prior to GWAS conditioning, and the middle pane shows SNP *P*-values after adjusting for the lead SNP (rs9976326). SNP shading denotes linkage disequilibrium with the lead SNP (r^2 = 0, white, r^2 = 1.0, dark red). SNPs (circles) plotted by GWAS *P*-values ($-\log_{10}$, left y-axis) and location (x-axis, GRCh37/hg19). Recombination rate (cM/Mb) on right y-axis, shown by light blue line. Lower section; ATAC-seq peaks showing chromatin accessability, shade denotes score. Multicolour bar shows chromatin states in 2 primary (E031 and E032) and one B-cell line (GM12878) generated using ChomHMM, see Supplementary Fig 7 for key.

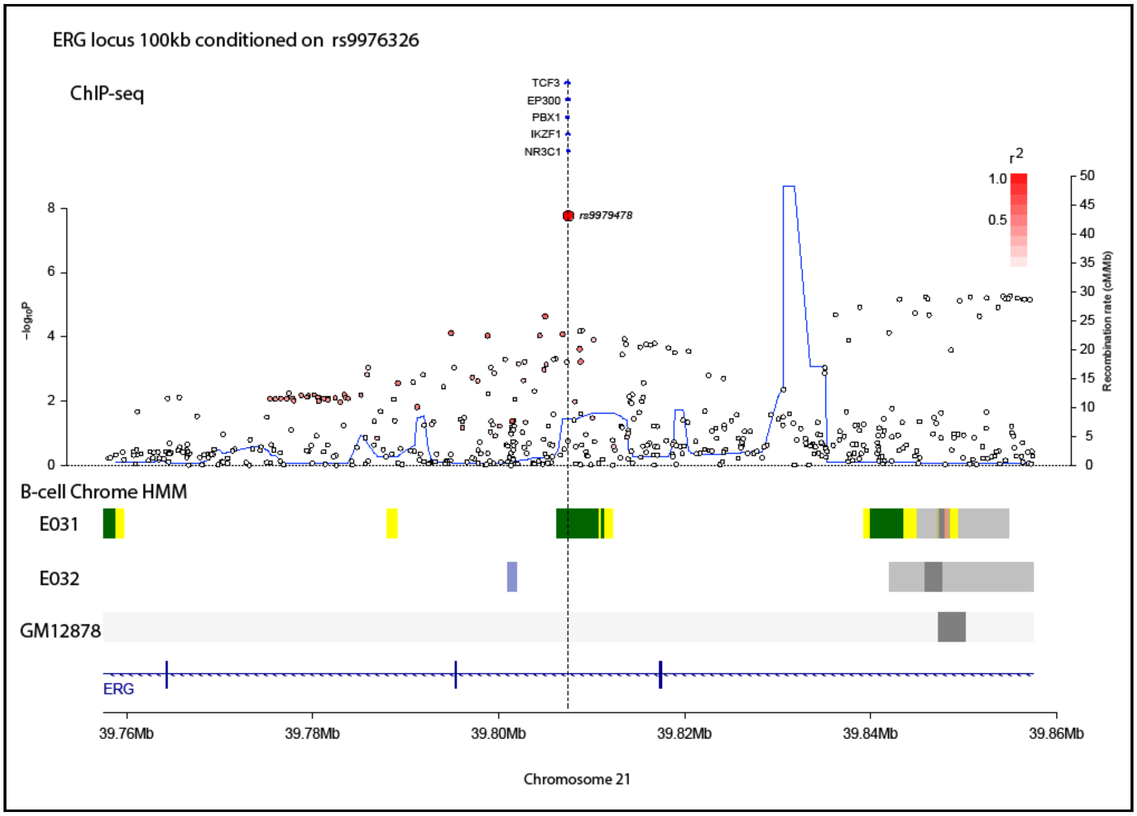

Supplementary Figrue 4: Regional association plot of 100kb window around the novel post conditioning SNP rs9975478 at chr21q22. Blue lines denote transcription factor ChIP-seq peaks in blood cells.

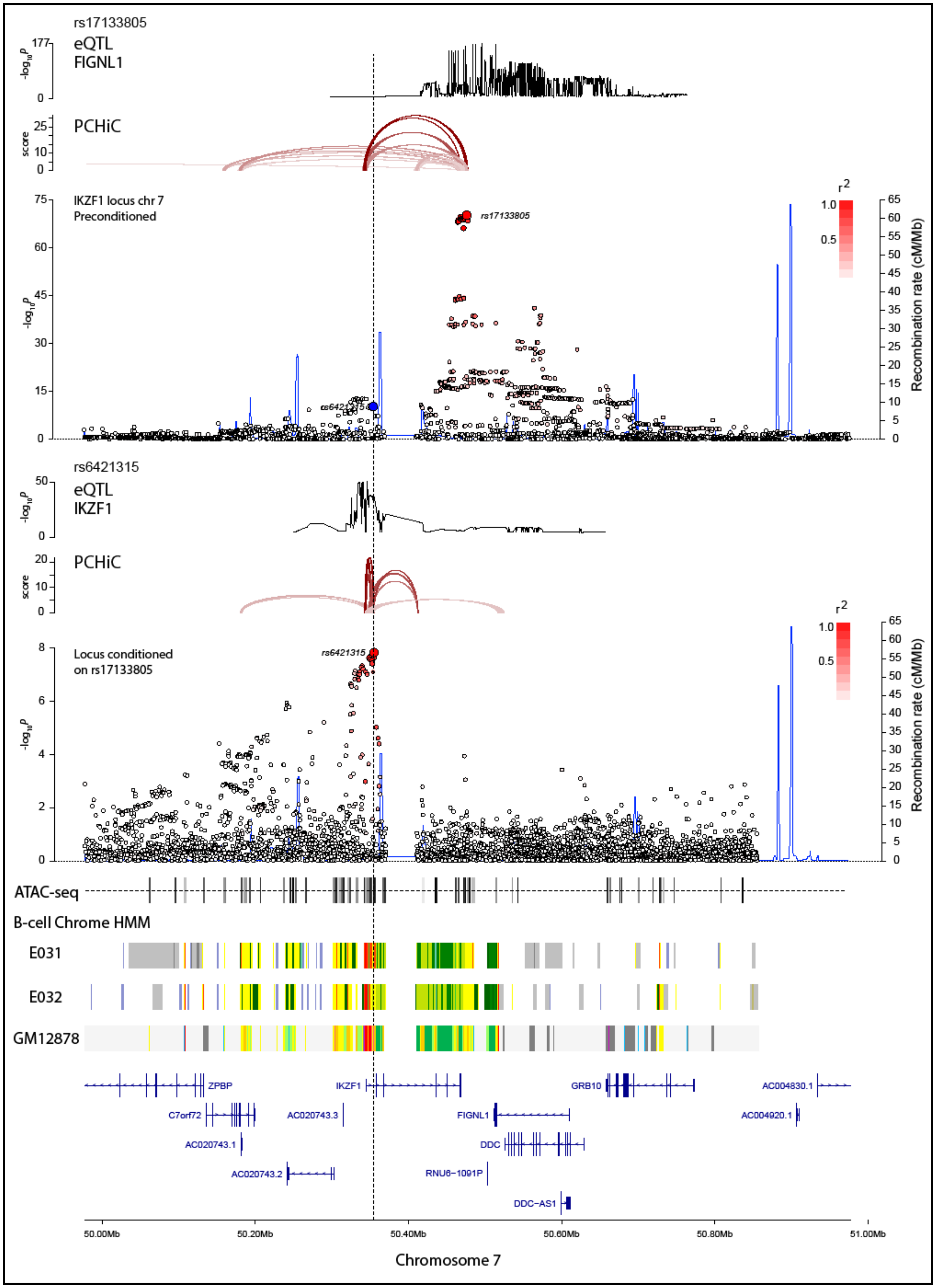

Supplementary Figrue 5: Pre and post conditional analysis association plots with epigenetic annoation. Top pane shows the chr7p12 locus prior to GWAS conditioning, and the middle pane shows SNP *P*-values after adjusting for the lead SNP (rs17133805). SNP shading denotes linkage

disequilibrium with the lead SNP ($r^2 = 0$, white, $r^2 = 1.0$, dark red). SNPs (circles) plotted by GWAS *P*-values ($-\log_{10}$, left y-axis) and location (x-axis, GRCh37/hg19). Recombination rate (cM/Mb) on right y-axis, shown by light blue line. EQTLs (black line) and looping promoter capture chromatin interactions (PCHiC, red arches) are shown for lead SNPs only in both pre and post conditional plots. Lead SNPs in each locus are defined as any SNP with a P-value < P(min) × 50 and $R^2 > 0.8$ from the lead SNP. Lower section; ATAC-seq peaks showing chromatin accessability, shade denotes score. Multicolour bar shows chromatin states in 2 primary (E031 and E032) and one B-cell line (GM12878) generated using ChomHMM, see Supplementary Fig 7 for key. Lower section; ATAC-seq peaks showing chromatin accessability, shade denotes score. Multicolour bar shows chromatin states in 2 primary (E031 and E032) and one B-cell line (GM12878) generated using ChomHMM, see Supp Fig 7 for ATAC scale and ChromHMM state definition.

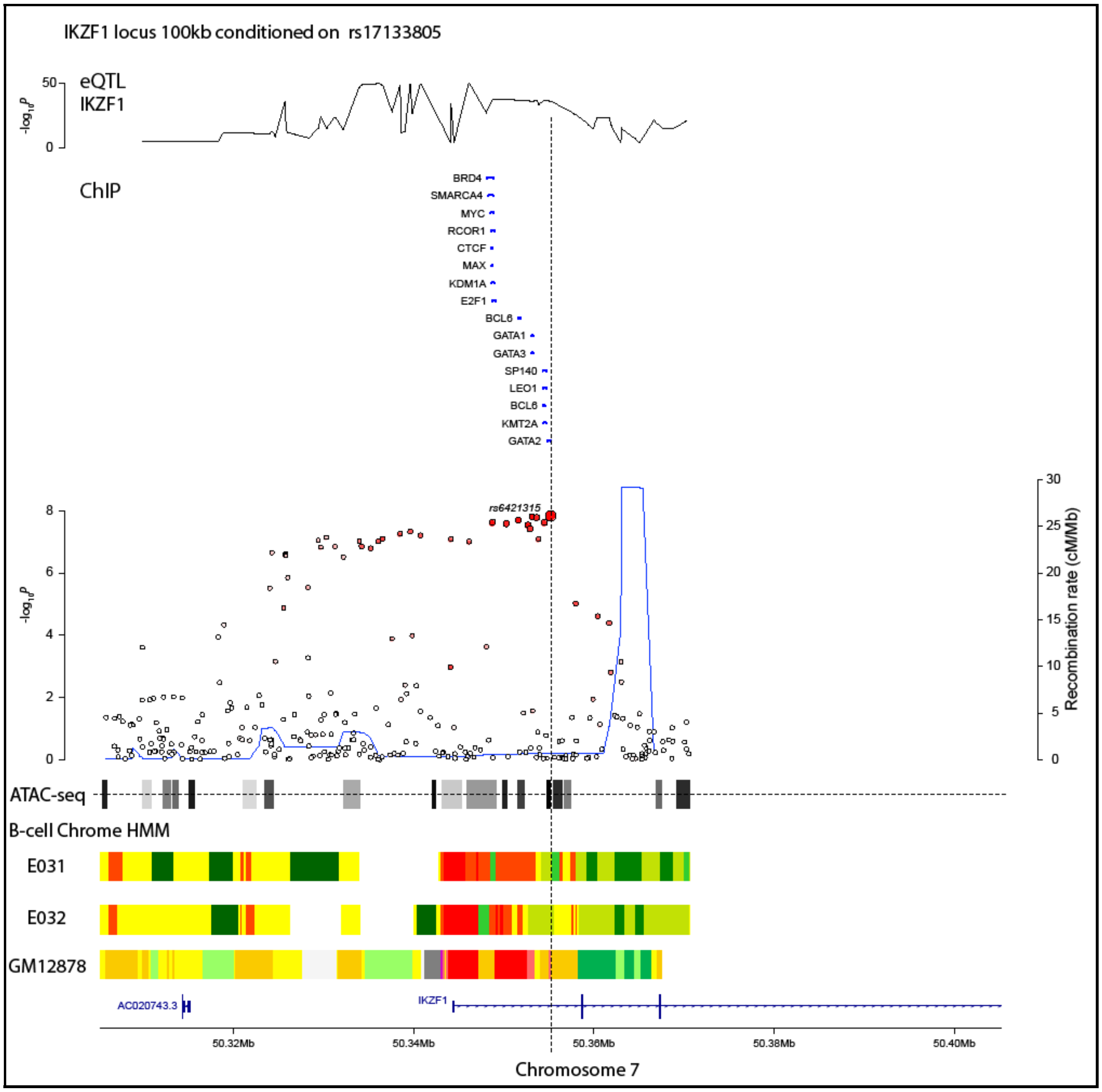

Supplementary Figrue 6: Regional association plot of 100kb window around the novel post conditioning SNP rs6421315 at chr21q22. Blue lines denote transcription factor ChIP-Seq peaks in blood cells.

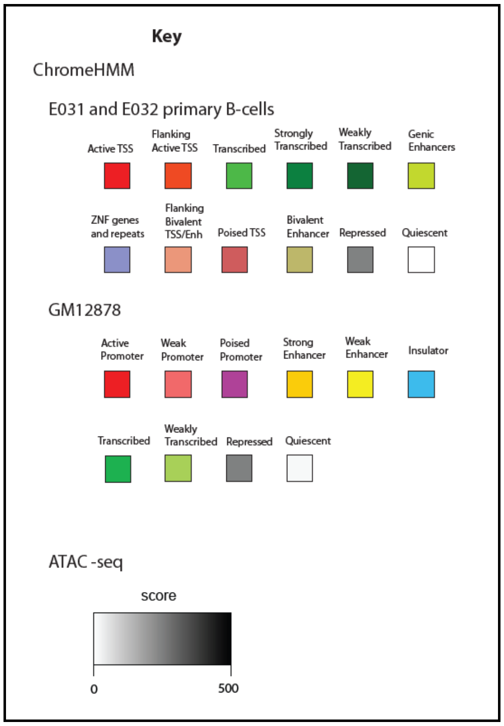

Supplementary Figure 7: Key for ATAC-seq and ChromHMM data ploted in Supplementary Figures 3,4,5 and 6.

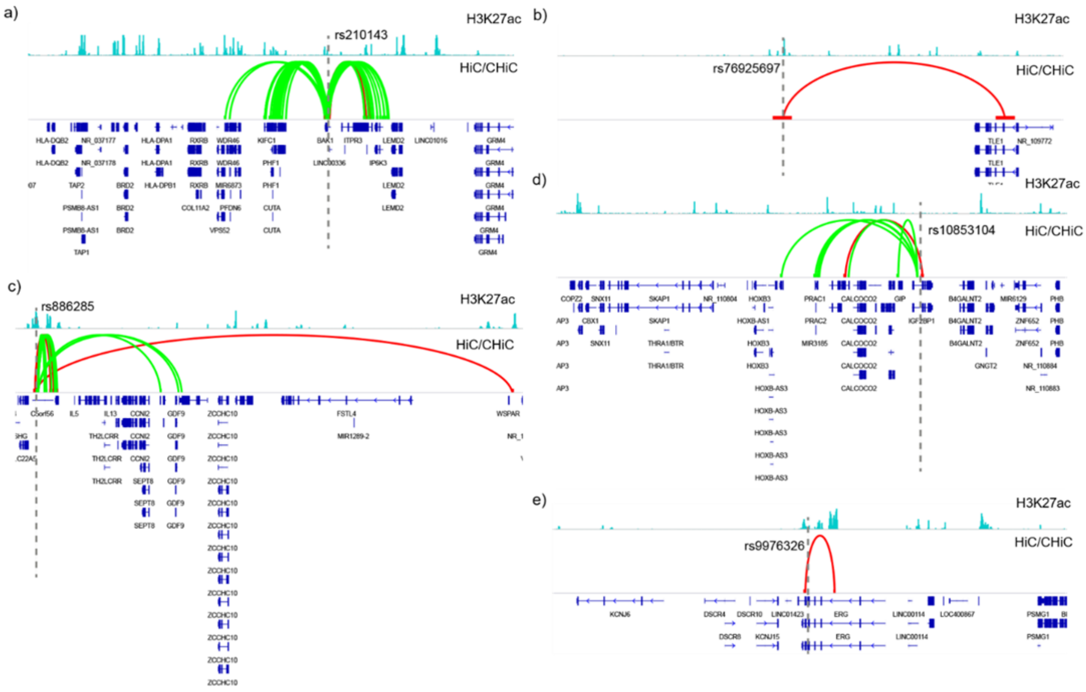

Supplementary Figure 8: Chromatin interaction plot of novel risk loci in human ALL cell line Nalm6 and naive B-cells. (a) for hyperdiploid ALL 6p21.31 (rs210143 in *BAK1*), (b) for B-ALL at 9q21.31 (rs76925697), (c) for hyperdiploid ALL at 5q31.1 (rs886285), (d) for *ETV6-RUNX1* ALL at 17q21.32 (rs10853104 in *IGF2BP1*) and (e) for *ERG* ALL at 21q22.2 (rs9976326). Histone acetylation mark and chromatin looping signals of Nalm6 were directly downloaded from the NCBI GEO GSE115494 dataset as described previously[1]. Loop interactions were call using HiCCUPS[2] from Juicer tools v1.12.01 under default parameters at resolution of 5000 and 10000 bp. Enriched interaction were reported with a false discovery rate < 0.1. For chromatin looping in Naïve B cells, promoter looping interactions were downloaded and filtered for a −log(weighted *P*) ≥ 5, as detrmined by the chromatin conformation capture assay (CHiC)[3]. Interactions were called using CHiCAGO[4]. Interactions overlapping lead SNPs in each locus are reported. ChIC singals from niave B-cells and HiC signals from ALL cell line Nalm6 are shown as green and red arches, respectively.

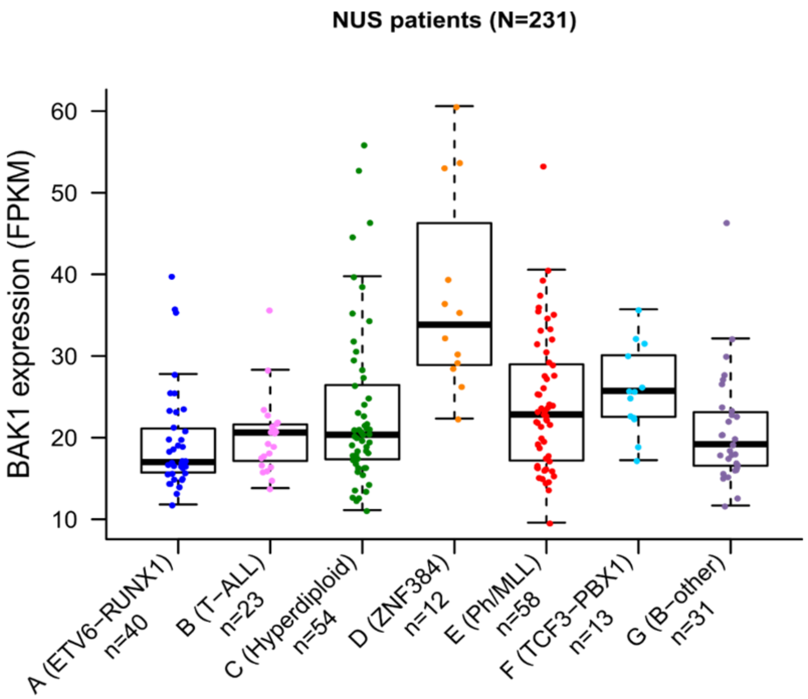

Supplementary Figure 9: Expression of the *BAK1* gene in different subgroups of ALL. There are seven different ALL subgroups identified by unsupervised hierarchical clustering of gene expression profile from 231 children with newly diagnosed ALL[5]. Each sample is represented by a dot and is colour-coded according to the subgroups it belongs to.

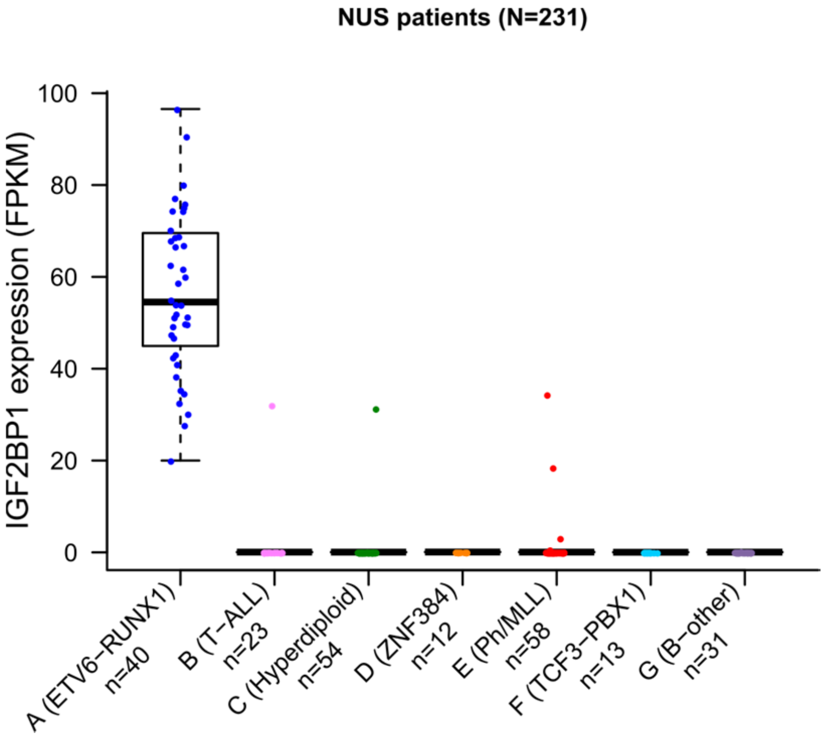

Supplementary Figure 10: Expression of the *IGF2BP1* gene in different subgroups of ALL. There are seven different ALL subgroups identified by unsupervised hierarchical clustering of gene expression profile from 231 children with newly diagnosed ALL[5]. Each sample is represented by a dot and is colour-coded according to the subgroups it belongs to. *ETV6-RUNX1* positive ALL cells showed significant overexpression of *IGF2BP1* compared to other ALL subtypes (P = 3.68 x 10^{-23}, by two-sided Wilcoxon rank sum test).

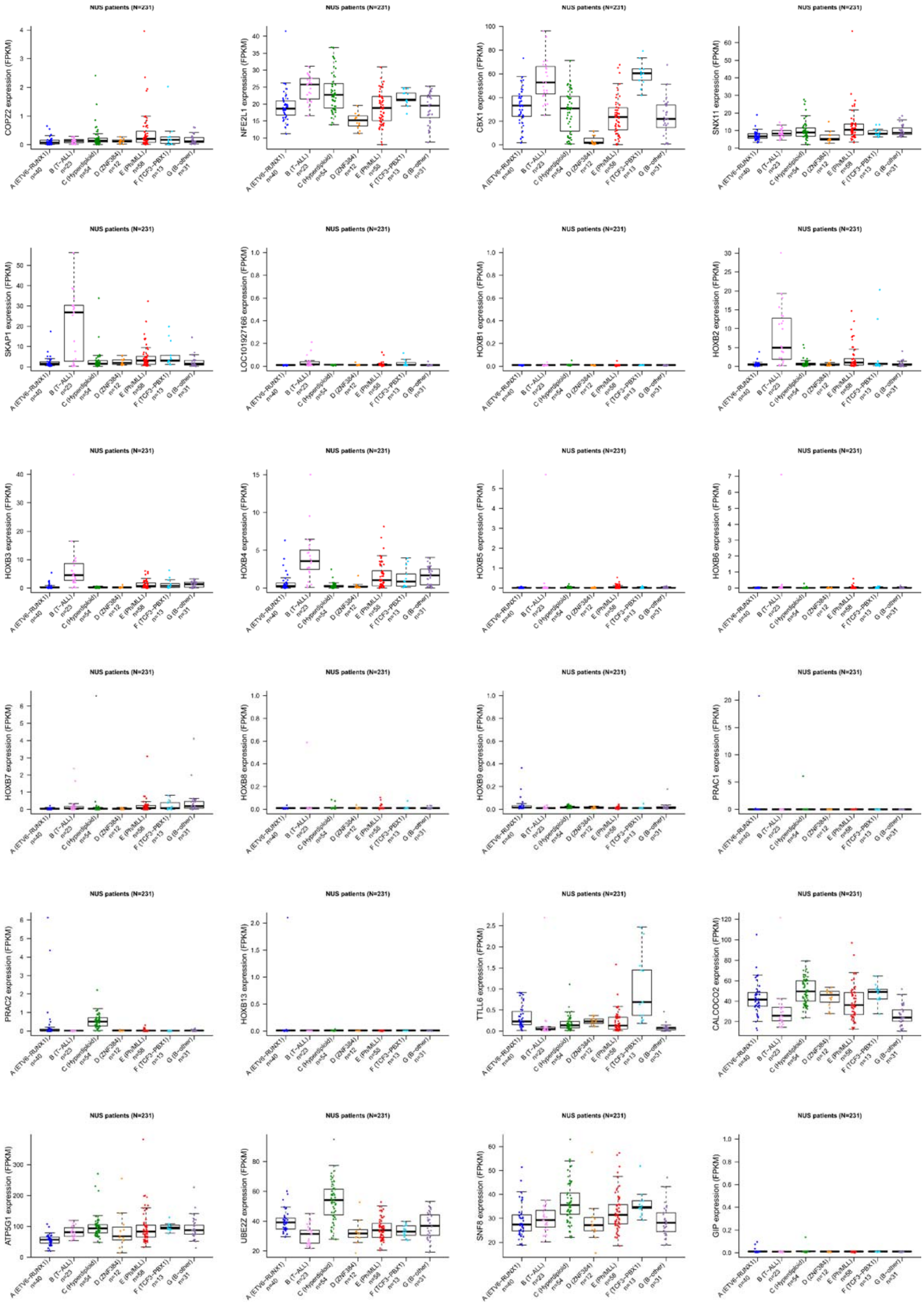
NUS patients (N=231)
COPZ2 expression (FPKM)
NFE2L1 expression (FPKM)
CBX1 expression (FPKM)
SNX11 expression (FPKM)
SKAP1 expression (FPKM)
LOC101927166 expression (FPKM)
HOXB1 expression (FPKM)
HOXB2 expression (FPKM)
HOXB3 expression (FPKM)
HOXB4 expression (FPKM)
HOXB5 expression (FPKM)
HOXB6 expression (FPKM)
HOXB7 expression (FPKM)
HOXB8 expression (FPKM)
HOXB9 expression (FPKM)
PRAC1 expression (FPKM)
PRAC2 expression (FPKM)
HOXB13 expression (FPKM)
TTLL6 expression (FPKM)
CALCOCO2 expression (FPKM)
ATP5G1 expression (FPKM)
UBE2Z expression (FPKM)
SNF8 expression (FPKM)
GIP expression (FPKM)
A (ETV6-RUNX1) n=40
B (T-ALL) n=23
C (Hyperdiploid) n=54
D (ZNF384) n=12
E (Ph/MLL) n=58
F (TCF3-PBX1) n=13
G (B-other) n=31

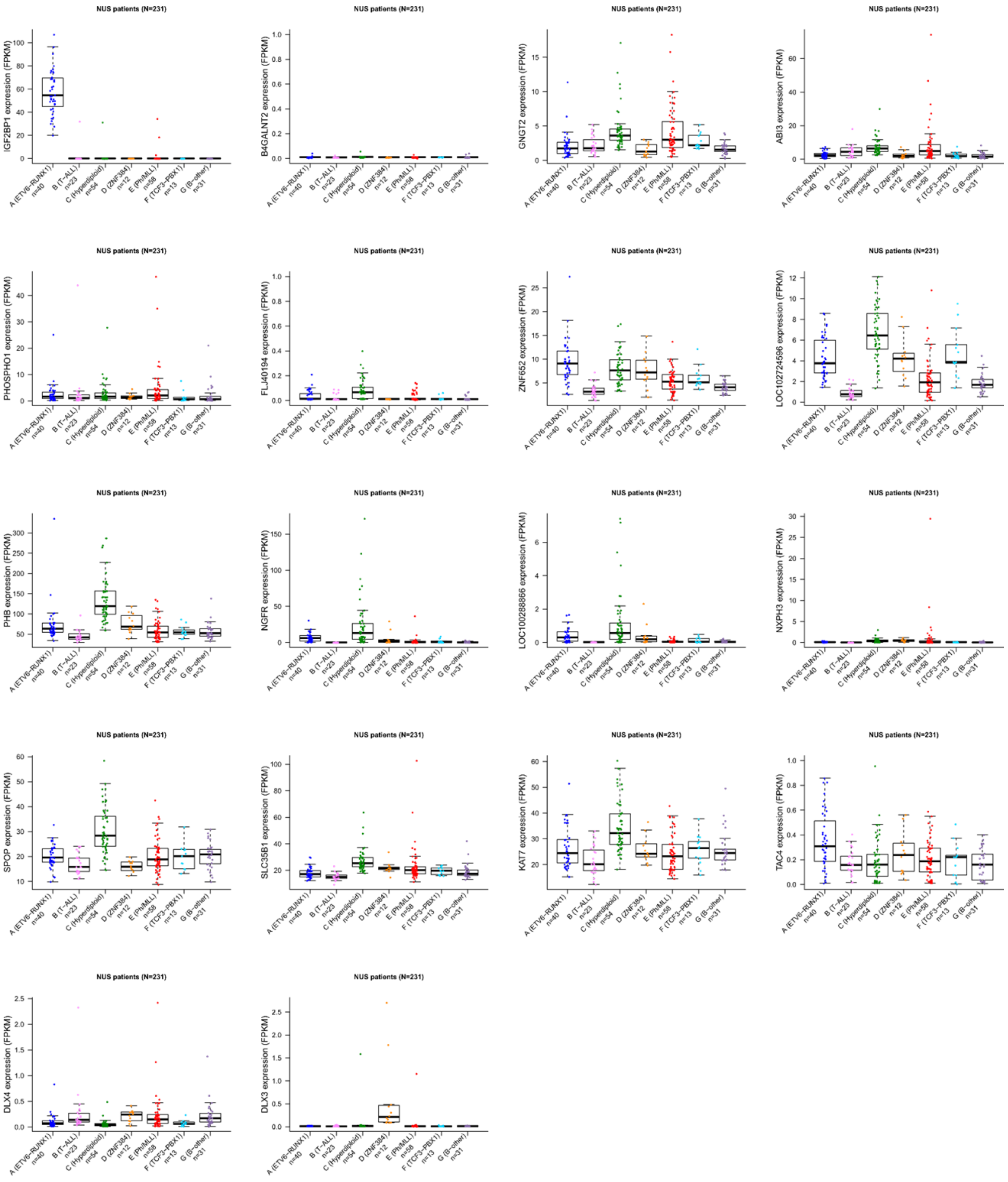

Supplementary Figure 11: Expression of all RefSeq genes within 1MB of the 17q12 locus in subgroups of ALL. There are seven different ALL subgroups identified by unsupervised hierarchical clustering of gene expression profile from 231 children with newly diagnosed ALL[5]. Each sample is represented by a dot and is colour-coded according to the subgroups it belongs to. No gene but *IGF2BP1* in this region was over-expressed specifically in the *ETV6-RUNX1* positive ALL subgroup.

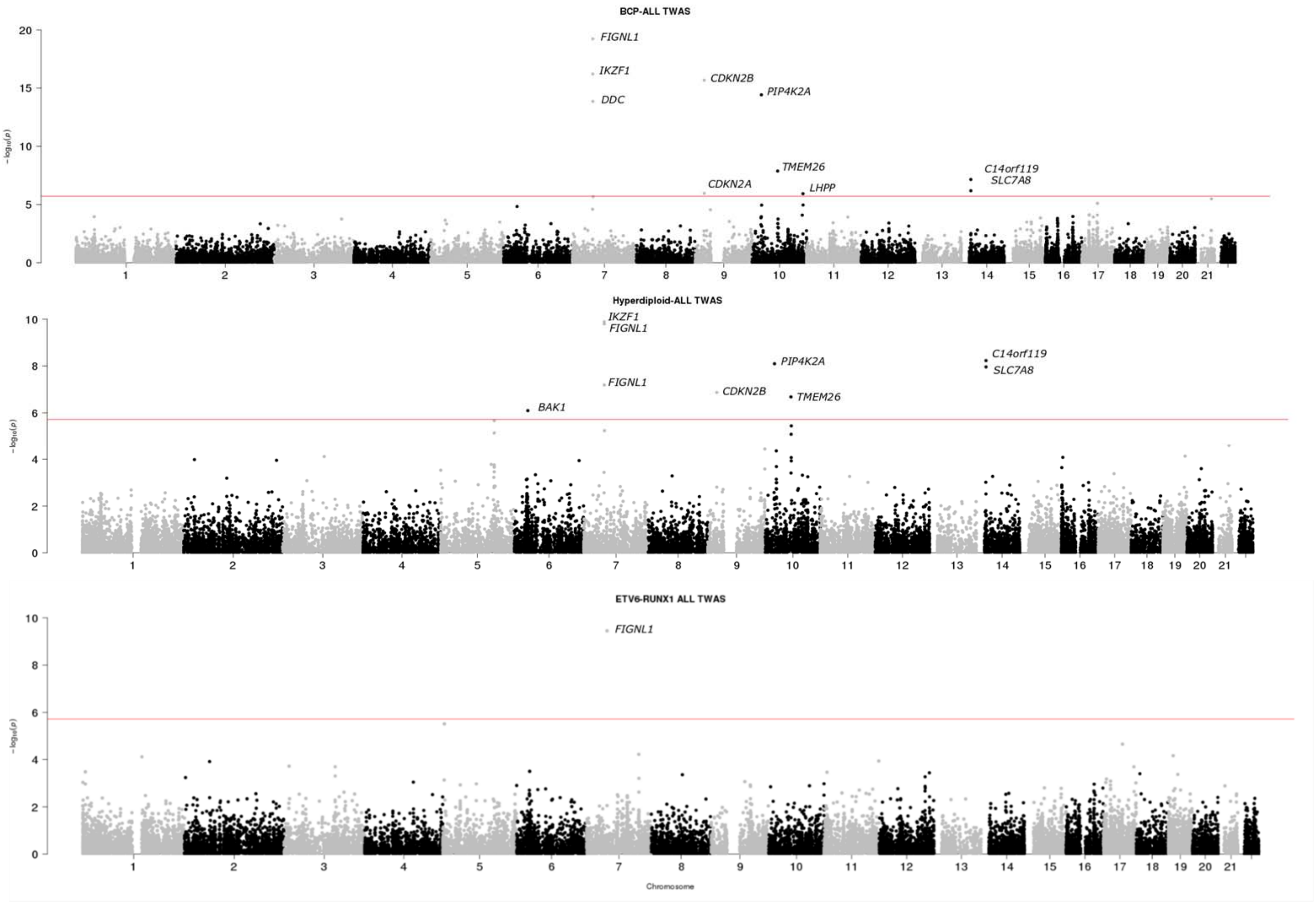

Supplementary Figrue 12: Reults from a multivariate anlaysis across 48 tissue eQTLs using summary data from B-ALL, high-hyperdiploid ALL and *ETV6-RUNX1* ALL using MulTiXcan. Red line indicates *P*-value threshold of 1.9×10^{-6}. On the X axis the chromosomes are shown while the Y-axis shows $-\log_{10}(P)$.

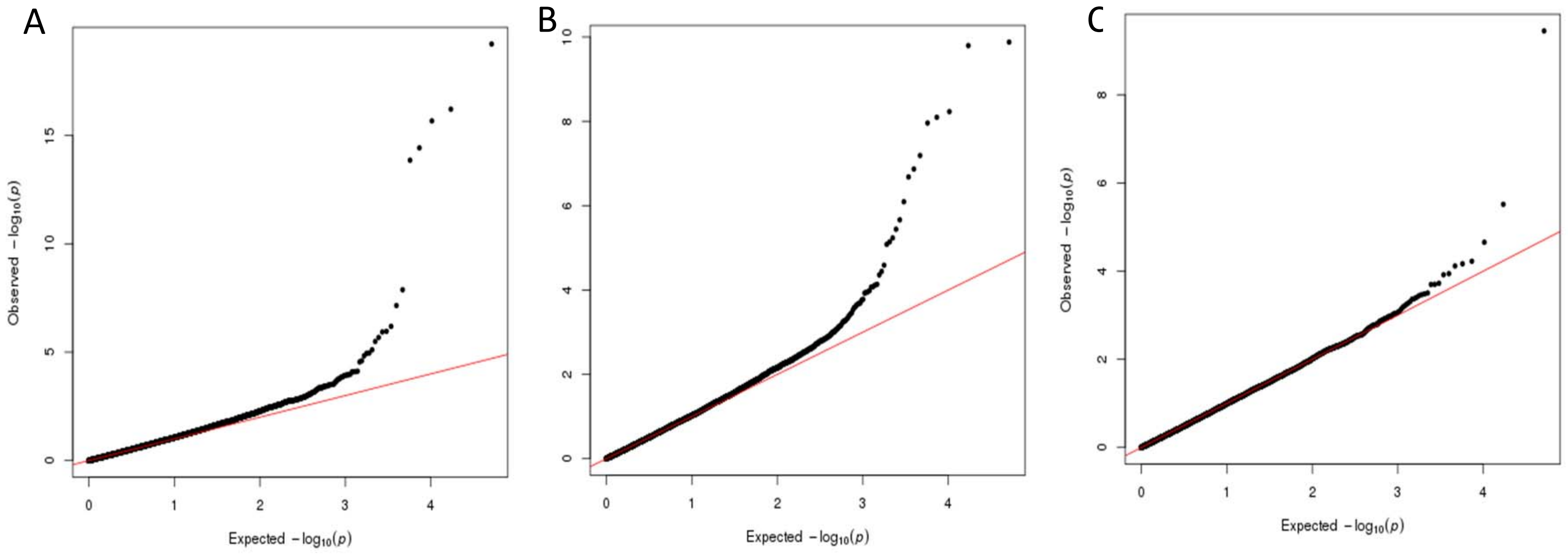

Supplementary Figure 13: QQ plots generated for the sMultixcan analysis for A: B-ALL, B: High-hyperdiploid ALL and C: *ETV6-RUNX1* positive ALL

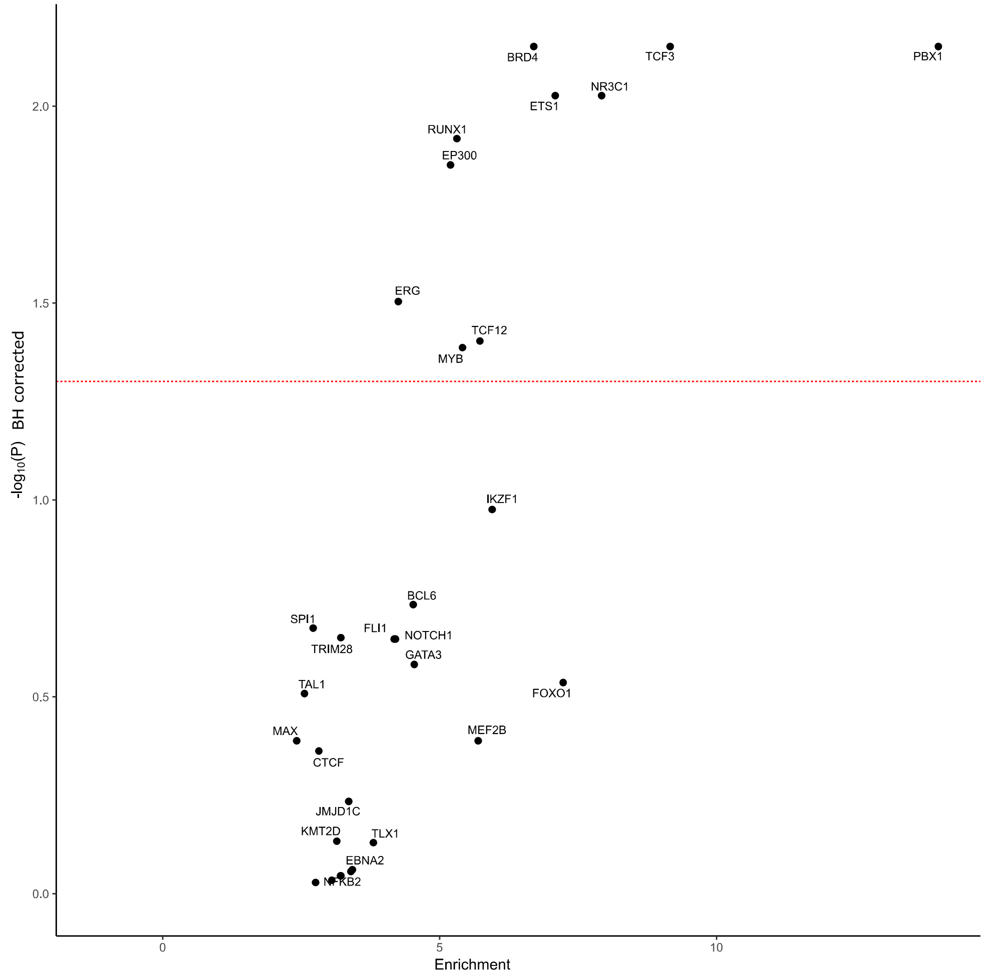

Supplementary Figrue 14: Enrichement of transcription factor binding at ALL risk loci. The frequnecy of transcription factor binding to risk SNPs (r^2 with lead SNP > 0.8) was tallied and compared to the frequency of binding to 10,000 random SNP premutations. The graph shows the fold enrichement (x-axis) and Benjamini–Hochberg corrected P-value (y-axis) performed for over 200 transcription factor binding profiles derived from ChIP experiements performed in blood related cell lines, extracted from ChIP-Atlas. Transcription factors with no enrichemnt are ommited.

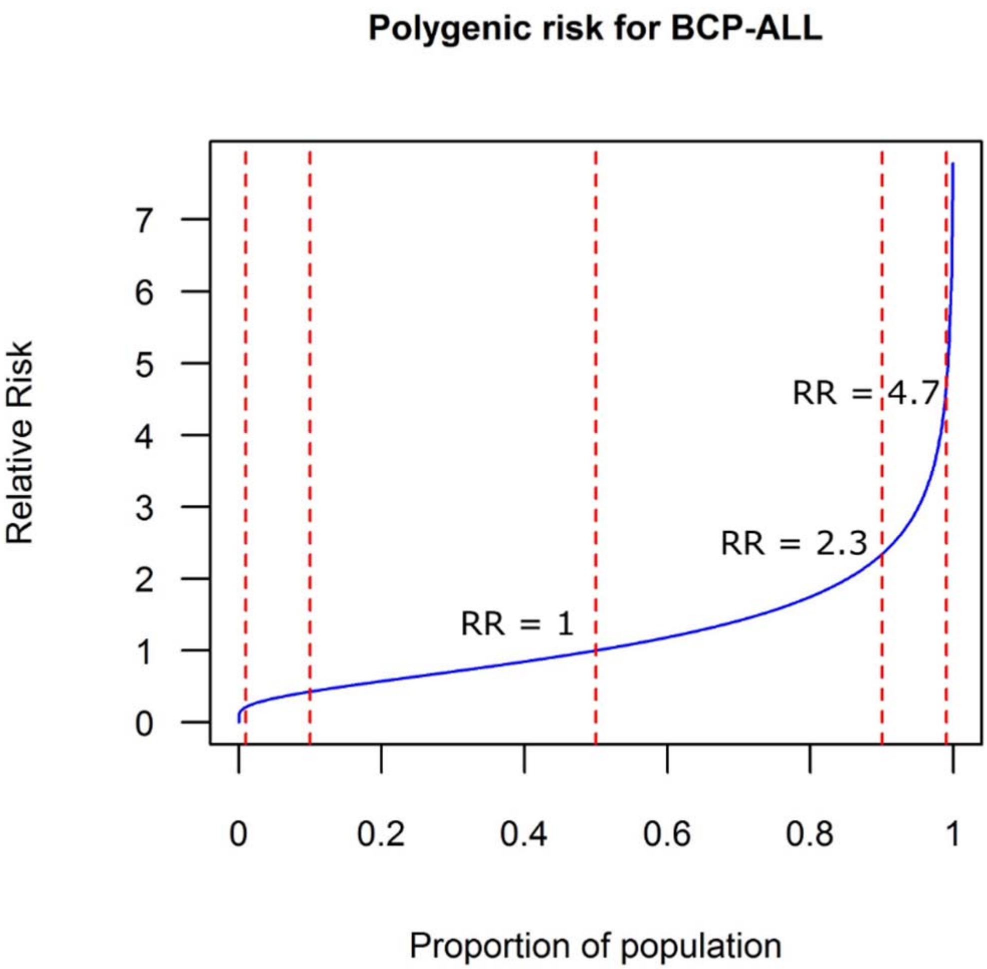

Supplementary Figrue 15: Distribution of ALL relative risk scores ordered by genetic risk. (risk is relative to population median risk). The blue line plots the distribution of relative risk (RR) across the population; the red lines correspond to 1st, 10th, 50th, 90th and 99th centiles. The RR figures presented in black are the average in the highest (i) 10th and (ii) 1 centile of genetic risk.

Supplementary Table 1: Summary of results for all risk SNPs. CHR: chromosome; BP: base pair; RAF: risk allele frequency; OR: odds ratio; CI: confidence intervals. OR and CI are derived from current meta-analysis.

CHR	SNP (Subtype)	Locus (gene)	Position (BP)	Risk Allele	RAF	OR(95% CI)	P-value
Top SNPs at previously published loci							
2	rs17481869 (*ETV6-RUNX1* postive)	2q22.3	146124454	A	0.08	1.74 (1.45-2.09)	2.37x10^{-09}
7	rs17133805	7p12.2 (*IKZF1*)	50477514	G	0.32	1.65 (1.56-1.74)	5.28x10^{-71}
8	rs75777619	8q24.21	130185176	G	0.12	1.26 (1.17-1.36)	2.30x10^{-09}
9	rs113650570	9p21.3 (*CDKN2A*)	21976402	A	0.02	2.32 (2.03-2.65)	8.06x10^{-35}
10	rs10821936	10q21.2 (*ARID5B*)	63723577	C	0.33	1.80 (1.71-1.89)	1.19x10^{-106}
10	rs3824662	10p14 (*GATA3*)	8104208	A	0.19	1.29 (1.21-1.38)	3.57x10^{-14}
10	rs2296624	10p12.2 (*PIP4K2A*)	22856946	C	0.67	1.25 (1.18-1.32)	2.79x10^{-15}
10	rs12779301	10q26.13 (*LHPP*)	126292655	C	0.66	1.22 (1.15-1.29)	5.72x10^{-13}
12	rs4762284	12q23.1 (*ELK3*)	96612762	T	0.32	1.15 (1.12-1.19)	3.75x10^{-07}
14	rs2239630	14q11.2 (*CEBPE*)	23589349	A	0.45	1.28 (1.22-1.35)	1.72x10^{-21}
21	rs9976326	21q22.2 (*ERG*)	39776485	T	0.25	1.19 (1.12-1.26)	1.11x10^{-08}
Top SNPs at novel genome-wide loci							
5	*rs11750693 (High-hyperdiploidy)	5q31.2 (near *KLHL3*)	137014631	C	0.02	2.62(1.90-3.61)	4.09x10^{-09}
5	rs886285 (High-hyperdiploidy)	5q31.1 (*C5orf56*)	131765206	T	0.34	1.29 (1.18-1.41)	1.56x10^{-08}
6	rs210143 (High-hyperdiploidy)	6p21.31 (*BAK1*)	33546930	C	0.73	1.30 (1.19-1.43)	2.21x10^{-08}
7	*rs74452384 (*ETV6-RUNX1* postive)	7q36.3 (near *UBE3C*)	156892518	G	0.01	3.97(2.43-6.46)	3.16x10^{-08}
9	rs76925697	9q21.31	83747371	A	0.96	1.52 (1.31-1.76)	2.11x10^{-08}
17	rs10853104 (*ETV6-RUNX1* postive)	17q21.32 (*IGF2BP1*)	47092076	T	0.47	1.33 (1.21-1.47)	1.82x10^{-08}
Novel subtype association							
21	rs9976326 (High-hyperdiploidy)	21q22.2 (*ERG*)	39776485	T	0.25	1.33 (1.21-1.46)	4.79x10^{-09}

*Not validated in replication cohort.

Supplementary Table 2: Results of validation of new genome-wide significant ALL risk loci in a replication cohort (COG-SJ-MESA-nonEA). BP: Base pair; RAF, risk allele frequency; OR:odds ratio; CI: confidence intervals; ALL: acute lymphoblastic leukaemia.

				**B-ALL (n = 2,237 vs 3,461)**			**High-hyperdipoidy (n = 422 vs 3,461)**			***ETV6-RUNX1* positive (n = 365 vs 3,461)**		
**SNP**	**Locus**	**Position (BP)**	**Risk Allele**	**RAF**	**OR (95% CI)**	***P*-value**	**RAF**	**OR (95% CI)**	***P*-value**	**RAF**	**OR (95% CI)**	***P*-value**
**<u>Validated</u>**												
rs76925697 (B-ALL)	9q21.31	83747371	A	0.98 vs 0.97	1.69 (1.28-2.25)	2.55x10^{-4}	0.99 vs 0.97	2.02 (1.15-3.52)	1.38x10^{-2}	0.98 vs 0.97	2.09 (1.15-3.79)	1.49x10^{-2}
rs886285 (High-hyperdiploidy)	5q31.1 (C5orf56)	131765206	T	0.43 vs 0.42	1.10 (1.00-1.21)	4.68x10^{-2}	0.45 vs 0.42	1.30 (1.10-1.52)	1.87x10^{-3}	0.43 vs 0.42	1.08 (0.91-1.28)	0.4
rs210143 (High-hyperdiploidy)	6p21.31 (*BAK1*)	33546930	C	0.76 vs 0.74	1.13 (1.02-1.25)	1.94x10^{-2}	0.77 vs 0.74	1.23 (1.03-1.49)	2.66x10^{-2}	0.73 vs 0.74	0.98 (0.78-1.10)	0.79
rs10853104 (ETV6-RUNX1 positive)	17q21.32 (*IGF2BP1*)	47092076	T	0.48 vs 0.46	1.05 (0.96-1.15)	0.27	0.47 vs 0.46	1.06 (0.91-1.25)	0.43	0.55 vs 0.46	1.36 (1.15-1.61)	2.96x10^{-4}
**<u>Not validated</u>**												
rs11750693 (High-hyperdiploidy)	5q31.2 (near *KLHL3*)	137014631	C	0.01 vs 0.01	0.99 (0.55-1.77)	0.98	0.01 vs 0.01	1.39 (0.57-3.39)	0.46	0.01 vs 0.01	1.15 (0.43-3.1)	0.77
rs74452384 (ETV6-RUNX1 positive)	7q36.3 (near *UBE3C*)	156892518	A	0.05 vs 0.06	0.78 (0.65-0.93)	6.00x10^{-3}	0.03 vs 0.06	0.63 (0.42-0.93)	2.18x10^{-2}	0.05 vs 0.06	0.77 (0.54-1.11)	0.16

Supplementary Table 3: Ancestry diversity in the replication series. Within the COG_SJ cohort, genetic ancestry (European [CEU], African [YRI], East Asian [JPT/CHB], and Native American) was determined by using ADMIXTURE (version 1.3.0)[6], with the sum of these 4 ancestries being 100% for any given subject. European American (EA), African American (AA), and Asian were defined as having >95% European genetic ancestry, >70% African ancestry, and >90% Asian ancestry, respectively. Hispanics were individuals for whom Native American ancestry was >10% and greater than African ancestry, as previously described[7]

Genetically-defined race	B-ALL	High-hyperdiploidy	ETV6-RUNX1 positive	Control (MESA)
Hispanics	1081 (48%)	190 (45%)	160 (44%)	682 (20%)
Africans	351 (16%)	57 (14%)	60 (16%)	1380 (40%)
Other	805 (36%)	175 (41%)	145 (40%)	1399 (40%)
In total	2237 (100%)	422 (100%)	365 (100%)	3461 (100%)

Supplementary Table 4: Genetic association with B-ALL, High-hyperdiploid and *ETV6-RUNX1* childhood ALL subtypes at novel ALL risk locus. P_{het}: P heterogeneity, I^2: Index to quantify dispersion of odds ratio, CI: Confidence Intervals.

**rs76925697 (A/T, 9q21.31)**	**Allele-T frequency**		**Sample numbers**				
**All B-ALL**	**Cases**	**Controls**	**Cases**	**Controls**	**OR**	**CI**	***P* -value**
UK GWAS I	0.02	0.03	824	5,200	0.56	(0.41-0.75)	$1.4x10^{-4}$
German GWAS	0.02	0.04	834	2,024	0.61	(0.43-0.86)	$5.3x10^{-3}$
UK GWAS II	0.03	0.04	784	7,385	0.73	(0.55-0.98)	$3.7x10^{-2}$
COG_SJ_MESA	0.02	0.04	2,879	2,057	0.71	(0.55-0.91)	$5.3x10^{-3}$
Total			5,321	16,666			
**Meta OR and *P* -value**					**0.66**	**(0.57-0.76)**	**$2.11x10^{-8}$**
						**P_{het}=0.53**	**I^2=0%**
**High-hyperdiploid**							
UK GWAS I	0.01	0.03	289	5,200	0.46	(0.29-0.74)	$1.5x10^{-3}$
German GWAS	0.02	0.04	176	2,024	0.54	(0.29-1.01)	$5.6x10^{-2}$
UK GWAS II	0.03	0.04	251	7,385	0.77	(0.46-0.73)	0.33
COG_SJ_MESA	0.02	0.04	653	2,057	0.60	(0.42-0.86)	$5.8x10^{-3}$
Total			1,369	16,666			
**Meta OR and *P* -value**					**0.58**	**(0.46-0.73)**	**$6.45x10^{-6}$**
						**P_{het}=0.58**	**I^2=0%**
***ETV6-RUNX1* positive**							
UK GWAS I	0.02	0.03	126	5,200	0.60	(0.30-1.22)	0.16
German GWAS	0.02	0.04	63	2,024	0.59	(0.20-1.71)	0.33
UK GWAS II	0.03	0.04	220	7,385	0.80	(0.56-0.97)	0.42
COG_SJ_MESA	0.03	0.04	527	2,057	0.77	(0.52-1.14)	0.19
Total			936	16,666			
**Meta OR and *P* -value**					**0.73**	**(0.56-0.97)**	**$3.02x10^{-2}$**
						**P_{het}=0.89**	**I^2=0%**

**rs9976326 (A/T,21q22.2, *ERG*)**	**Allele-T frequency**		**Sample numbers**				
**All B-ALL**	**Cases**	**Controls**	**Cases**	**Controls**	**OR**	**CI**	***P* -value**
UK GWAS I	0.28	0.25	824	5,200	1.20	(1.06-1.26)	$4.1x10^{-3}$
German GWAS	0.30	0.26	834	2,024	1.22	(1.06-1.41)	$6.1x10^{-3}$
UK GWAS II	0.28	0.25	784	7,385	1.18	(1.04-1.33)	$9.3x10^{-3}$
COG_SJ_MESA	0.29	0.25	2,879	2,057	1.17	(1.06-1.29)	$1.3x10^{-3}$
Total			5,321	16,666			
**Meta OR and *P* -value**					**1.19**	**(1.12-1.26)**	**$1.11x10^{-8}$**
						**P_{het}=0.97**	**I^2=0%**
**High-hyperdiploid**							

UK GWAS I	0.33	0.25	289	5,200	1.61	(1.32-1.96)	$2.22x10^{-6}$
German GWAS	0.30	0.26	176	2,024	1.22	(0.93-1.59)	0.15
UK GWAS II	0.30	0.25	251	7,385	1.32	(1.07-1.64)	$1x10^{-2}$
COG_SJ_MESA	0.29	0.25	653	2,057	1.23	(1.07-1.42)	$4.1x10^{-3}$
Total			1,369	16,666			
**Meta OR and *P* -value**					**1.33**	**(1.21-1.46)**	**$4.79x10^{-9}$**
						**P_{het}=0.17**	**I^2=41%**
***ETV6-RUNX1* positive**							
UK GWAS I	0.26	0.25	126	5,200	1.07	(0.80-1.44)	0.66
German GWAS	0.26	0.26	63	2,024	0.99	(0.64-1.54)	0.98
UK GWAS II	0.24	0.25	220	7,385	0.92	(0.73-1.15)	0.45
COG_SJ_MESA	0.24	0.25	527	2,057	0.94	(0.80-1.10)	0.42
Total			936	16,666			
**Meta OR and *P* -value**					**0.95**	**(0.85-1.07)**	**0.42**
						**P_{het}=0.85**	**I^2=0%**

**rs886285 (C/T, 5q31.1, *C5orf56*)**	**Allele-T frequency**		**Sample numbers**				
**All B-ALL**	**Cases**	**Controls**	**Cases**	**Controls**	**OR**	**CI**	***P* -value**
UK GWAS I	0.33	0.31	824	5,200	0.94	(0.84-1.05)	0.27
German GWAS	0.38	0.34	834	2,024	0.84	(0.74-0.96)	$9.7x10^{-3}$
UK GWAS II	0.35	0.31	784	7,385	0.83	(0.74-0.93)	$9.0x10^{-4}$
COG_SJ_MESA	0.33	0.32	2,879	2,057	0.96	(0.87-1.05)	0.37
Total			5,321	16,666			
**Meta OR and *P* -value**					**0.90**	**(0.85-0.95)**	**$1.6x10^{-4}$**
						**P_{het}=0.14**	**I^2=45%**
**High-hyperdiploid**							
UK GWAS I	0.34	0.31	289	5,200	0.88	(0.73-1.05)	$9.2x10^{-2}$
German GWAS	0.43	0.34	176	2,024	0.68	(0.54-0.86)	$1.4x10^{-3}$
UK GWAS II	0.41	0.31	251	7,385	0.64	(0.53-0.77)	$4.6x10^{-6}$
COG_SJ_MESA	0.36	0.32	653	2,057	0.83	(0.73-0.96)	$9.1x10^{-3}$
Total			1,369	16,666			
**Meta OR and *P* -value**					**0.78**	**(0.71-0.85)**	**$1.56x10^{-8}$**
						**P_{het}=0.05**	**I^2=62%**
***ETV6-RUNX1* positive**							
UK GWAS I	0.32	0.31	126	5,200	0.98	(0.75-1.28)	0.87
German GWAS	0.28	0.34	63	2,024	1.24	(1.84-1.84)	0.28
UK GWAS II	0.34	0.31	220	7,385	0.90	(0.73-1.10)	0.31
COG_SJ_MESA	0.32	0.32	527	2,057	1.0	(0.85-1.17)	0.98
Total			936	16,666			
**Meta OR and *P* -value**					**0.98**	**(0.88-1.10)**	**0.43**
						**P_{het}=0.55**	**I^2=0%**

**rs210143 (C/T, 6p21.31,**	**Allele-T**		**Sample**				

BAK1)	frequency		numbers				
**All B-ALL**	**Cases**	**Controls**	**Cases**	**Controls**	**OR**	**CI**	***P* -value**
UK GWAS I	0.25	0.31	824	5,200	1.32	(1.10-1.58)	7.3x10^{-2}
German GWAS	0.22	0.28	834	2,024	1.30	(1.01-1.68)	7.0x10^{-3}
UK GWAS II	0.22	0.30	784	7,385	1.46	(1.18-1.81)	5.1x10^{-3}
COG_SJ_MESA	0.24	0.28	2,879	2,057	1.13	(1.02-1.24)	1.63x10^{-2}
Total			5,321	16,666			
**Meta OR and *P* -value**					**1.15**	**(1.08-1.22)**	**1.74x10^{-6}**
						**P_{het}=0.74**	**I^2=0%**

**High-hyperdiploid**							
UK GWAS I	0.25	0.31	289	5,200	1.32	(1.10-1.58)	2.97x10^{-3}
German GWAS	0.22	0.28	176	2,024	1.30	(1.01-1.68)	4.03x10^{-2}
UK GWAS II	0.22	0.30	251	7,385	1.46	(1.18-1.81)	3.22x10^{-4}
COG_SJ_MESA	0.24	0.28	653	2,057	1.23	(1.06-1.42)	1.63x10^{-2}
Total			1,369	16,666			
**Meta OR and *P* -value**					**1.30**	**(1.19-1.43)**	**2.21x10^{-8}**
						**P_{het}=0.62**	**I^2=0%**

***ETV6-RUNX1* positive**							
UK GWAS I	0.34	0.31	126	5,200	0.86	(0.66-1.13)	0.27
German GWAS	0.21	0.28	63	2,024	1.43	(0.95-2.16)	8.94x10^{-2}
UK GWAS II	0.29	0.30	220	7,385	1.01	(0.82-1.24)	0.93
COG_SJ_MESA	0.27	0.28	527	2,057	1.06	(0.90-1.24)	0.48
Total			936	16,666			
**Meta OR and *P* -value**					**1.03**	**(0.92-1.15)**	**0.59**
						**P_{het}=**	**I^2=0%**

**rs10853104 (T/C, 17q21.32, *IGF2BP1*)**	**Allele-C frequency**		**Sample numbers**				
**All B-ALL**	**Cases**	**Controls**	**Cases**	**Controls**	**OR**	**CI**	***P* –value**
UK GWAS I	0.49	0.50	824	5,200	1.03	(0.92-1.14)	0.64
German GWAS	0.49	0.49	834	2,024	0.99	(0.87-1.11)	0.82
UK GWAS II	0.47	0.48	784	7,385	1.07	(0.96-1.19)	0.20
COG_SJ_MESA	0.48	0.49	2,879	2,057	1.06	(0.98-1.16)	0.16
Total			5,321	16,666			
**Meta OR and *P* -value**					**1.04**	**(0.99-1.10)**	**0.11**
						**P_{het}=0.71**	**I^2=0%**

**High-hyperdiploid**							
UK GWAS I	0.52	0.50	289	5,200	0.91	(0.77-1.08)	0.28
German GWAS	0.53	0.51	176	2,024	0.92	(0.74-1.16)	0.50
UK GWAS II	0.49	0.48	251	7,385	0.98	(0.81-1.17)	0.78
COG_SJ_MESA	0.50	0.49	653	2,057	0.98	(0.87-1.12)	0.82
Total			1,369	16,666			
**Meta OR and *P* -value**					**0.96**	**(0.88-1.04)**	**0.29**

						**P_{het}=0.88**	**I^2=0%**
***ETV6-RUNX1* positive**							
UK GWAS I	0.43	0.50	126	5,200	1.32	(1.02-1.69)	$3.18x10^{-2}$
German GWAS	0.41	0.51	63	2,024	1.52	(1.05-2.21)	$2.60x10^{-2}$
UK GWAS II	0.45	0.48	220	7,385	1.15	(0.95-1.40)	0.14
COG_SJ_MESA	0.40	0.49	527	2,057	1.42	(1.23-1.64)	$1.28x10^{-6}$
Total			936	16,666			
**Meta OR and *P* -value**					**1.33**	**(1.21-1.47)**	**$1.82x10^{-8}$**
						**P_{het}=0.33**	**I^2=13%**

Supplementary Table 5: Genotype counts of risk alleles for the individual cohorts post imputation.

SNP(Type)	Genotype count					
**rs76925697 (9q21.31, B-ALL)**	**AA**	**AT**	**TT**	**AA**	**AT**	**TT**
UK GWAS I	797	26	1	4843	354	4
German GWAS	798	35	1	1879	141	4
UKGWAS II	742	42	0	6854	522	10
COG-SJ_MESA	2743	135	1	1916	138	3
**rs9976326 (*ERG*, B-ALL)**	**AA**	**AT**	**TT**	**AA**	**AT**	**TT**
UK GWAS I	429	332	63	2969	1912	318
German GWAS	401	366	67	1082	828	114
UKGWAS II	410	304	71	4093	2818	474
COG-SJ_MESA	1500	1143	235	1179	734	144
**rs9976326 (*ERG*, High-hyperdiploidy)**	**AA**	**AT**	**TT**	**AA**	**AT**	**TT**
UK GWAS I	128	131	30	2969	1912	318
German GWAS	85	77	14	1082	828	114
UKGWAS II	121	108	22	4093	2818	474
COG-SJ_MESA	325	271	57	1179	734	144
**rs886285 (*C5orf56*, High-hyperdiploidy)**	**CC**	**CT**	**TT**	**CC**	**CT**	**TT**
UK GWAS I	39	118	131	497	2257	2446
German GWAS	34	82	60	238	911	875
UKGWAS II	43	120	88	745	3144	3495
COG-SJ_MESA	92	287	274	204	914	939
**rs210143 (BAK1, High-hyperdiploidy)**	**CC**	**CT**	**TT**	**CC**	**CT**	**TT**
UK GWAS I	16	111	162	500	2186	2514
German GWAS	12	55	109	154	836	1034
UKGWAS II	13	86	152	648	3063	3673
COG-SJ_MESA	40	231	382	157	821	1079
**rs10853104 (*IGF2BP1, ETV6-RUNX1*)**	**TT**	**CT**	**CC**	**TT**	**CT**	**CC**
UK GWAS I	25	58	43	1285	2604	1311
German GWAS	10	31	22	525	1004	494
UKGWAS II	49	100	71	1694	3751	1940
COG-SJ_MESA	86	252	189	505	1022	530

Supplementary Table 6: Conditional analysis on the most significant SNP at all previously identified and novel risk loci within a one MB window. CHR: chromosome; BP: base pair; RAF: risk allele frequency; OR: odds ratio; CI: confidence intervals. OR and CI are derived from current meta-analysis.

CHR	Top SNP at locus (conditioned on)	*P-value*	Locus (gene)	Position (BP)	Risk Allele	Post-conditional top SNP	*P-value* (Post-conditional)	*P-value* (Pre-conditional)
**B-ALL**								
7	rs17133805	5.28×10^{-71}	7p12.2 (*IKZF1*)	50477514	G	rs6421315	1.43×10^{-08}	5.28×10^{-11}
8	rs75777619	2.30×10^{-09}	8q24.21	130185176	G	rs4733633	3.93×10^{-04}	6.70×10^{-04}
9	rs113650570	8.06×10^{-35}	9p21.3 (*CDKN2A*)	21976402	A	rs944800	4.05×10^{-08}	4.12×10^{-07}
9	*rs76925697	2.11×10^{-08}	9q21.31	83747371	A	rs2796471	3.12×10^{-05}	8.04×10^{-05}
10	rs10821936	1.19×10^{-106}	10q21.2 (*ARID5B*)	63723577	C	rs2893901	3.20×10^{-03}	1.09×10^{-02}
10	rs3824662	3.57×10^{-14}	10p14 (*GATA3*)	8104208	A	rs11255520	5.36×10^{-04}	2.68×10^{-02}
10	rs2296624	2.79×10^{-15}	10p12.2 (*PIP4K2A*)	22856946	C	rs74229974	1.33×10^{-08}	1.33×10^{-08}
10	rs12779301	5.72×10^{-13}	10q26.13 (*LHPP*)	126292655	C	rs11245326	4.90×10^{-04}	1.78×10^{-07}
14	rs2239630	1.72×10^{-21}	14q11.2 (*CEBPE*)	23589349	A	rs56031127	2.08×10^{-04}	1.35×10^{-14}
21	rs9976326	1.11×10^{-08}	21q22.2 (*ERG*)	39776485	T	rs9979478	1.69×10^{-08}	1.89×10^{-08}
ETV6-RUNX1								
2	rs17481869	2.37×10^{-09}	2q22.3	146124454	A	rs13384504	2.06×10^{-02}	8.30×10^{-04}
17	*rs10853104	1.82×10^{-08}	17q21.32 (*IGF2BP1*)	47092076	T	rs12451571	9.69×10^{-04}	5.88×10^{-04}
**High-hyperdiploidy**								
5	*rs886285	1.56×10^{-08}	5q31.1 (*C5orf56*)	131765206	T	rs28435738	8.26×10^{-05}	1.86×10^{-02}
6	*rs210143	2.21×10^{-08}	6p21.31 (*BAK1*)	33546930	C	rs9461938	6.95×10^{-04}	1.59×10^{-03}
21	rs9976326	4.79×10^{-09}	21q22.2 (*ERG*)	39776485	T	rs9979478	9.37×10^{-07}	1.05×10^{-06}
10	rs10821936	1.71×10^{-96}	10q21.2 (*ARID5B*)	63723577	C	rs76420690	5.43×10^{-04}	3.87×10^{-06}

*New loci discovered in current meta-analyses.

Supplementary Table 7: Conditional analysis on the 21q22.2 locus (B-ALL)

**SNP (gene)**	**Meta-analysis *P*-value**	**Conditioned on rs9976326**	**Conditioned on rs9979478**
rs9976326 (*ERG*)	1.11×10^{-08}	**-**	**1.03×10^{-08}**
rs9979478 (*ERG*)	1.89×10^{-08}	**1.69×10^{-08}**	**-**

Supplementary Table 8: Lead SNPs used for filtering CHiC and ChIP data. CHR: chromosome; POS: position in basepair. *P*-value shown from the current meta-analysis.

Locus	SNP	Distance from lead SNP	r^2	CHR	POS (Hg19)	*P*-value	Disease Subset
6p21 (*BAK1*)	**rs210143**	**0**	**1**	**6**	**33546930**	**2.21E-08**	HD
	rs210142	-93	1	6	33546837	2.34E-08	HD
	rs210134	-6721	0.8956	6	33540209	3.17E-07	HD
5q31 (*IRF1*)	**rs886285**	**0**	**1**	**5**	**131765206**	**1.56E-08**	HD
	rs2522055	35393	0.9264	5	131800599	2.83E-08	HD
	rs2706387	24351	0.9264	5	131789557	3.96E-08	HD
	rs6865438	25210	0.9264	5	131790416	4.02E-08	HD
	rs7703230	21402	0.9349	5	131786608	4.06E-08	HD
	rs4475253	11300	0.9736	5	131776506	4.12E-08	HD
	rs726923	21212	0.9391	5	131786418	4.30E-08	HD
	rs12659708	24657	0.9264	5	131789863	5.02E-08	HD
	rs2522043	26465	0.9264	5	131791671	5.05E-08	HD
	rs12655512	24539	0.9264	5	131789745	5.28E-08	HD
	rs2522044	26640	0.9264	5	131791846	5.43E-08	HD
	rs4504381	20564	0.9391	5	131785770	5.56E-08	HD
	rs10059611	22072	0.9307	5	131787278	6.13E-08	HD
	rs7713818	23545	0.9219	5	131788751	9.24E-08	HD
	rs6866467	21847	0.8709	5	131787053	1.90E-07	HD
21q22 (*ERG*)	**rs9976326**	**0**	**1**	**21**	**39776485**	**4.79E-09**	**HD**
	rs55681902	8267	0.9681	21	39784752	2.43E-08	HD
17q12 (*IGF2BP1*)	**rs10853104**	**0**	**1**	**17**	**47092076**	**1.82E-08**	**ETV6-RUNX1**
9q21	**rs76925697**	**0**	**1**	**9**	**83747371**	**2.11E-08**	**Generic**
	rs62579834	453	1	9	83747824	2.12E-08	Generic
	rs62579836	5567	0.9746	9	83752938	9.03E-08	Generic
	rs62579838	7695	0.9746	9	83755066	1.21E-07	Generic
	rs62579841	10202	0.9746	9	83757573	1.18E-07	Generic
	rs868524	14994	0.9493	9	83762365	1.13E-07	Generic

Top SNP in each locus shown in bold. HD = High-hyperdiploid ALL

Supplementary Table 9: CHiC contacts at risk loci. Lead SNPs at each locus used for filtering CHiC and ChIP data defined by a *P*-value < *P*(min) × 50 and $R^2 > 0.8$ from the top SNP. Contact scores: CHiCAGO scores with significance (≥5) as per[4].

Locus: rs10853104 chr17 (*IGF2BP1*)

SNP	SNP bin start	SNP bin end	Promoter bin start	Promoter bin end	Contact score	Gene TSS
**rs10853104**	47089144	47092652	46701021	46706541	7.6	*HOXB9,HOXB-AS4*
**rs10853104**	47089144	47092652	46795626	46803741	10.3	*MIR3185,PRAC1, PRAC2*
**rs10853104**	47089144	47092652	46803742	46807447	11.0	*HOXB13*
**rs10853104**	47089144	47092652	46807448	46814524	8.3	*CTD-2377D24.4*
**rs10853104**	47089144	47092652	46892929	46896186	12.0	*TTLL6*

Locus: rs886285 chr5 (*C5orf56*)

SNP	SNP bin start	SNP bin end	Promoter bin start	Promoter bin end	Contact score	Gene TSS
**rs2522043, rs2522044**	131744512	131749741	131791495	131792921	5.4	*C5orf56*
**rs886285**	131758776	131769186	131784623	131785167	5.5	
**rs886285, rs2522043, rs2522044**	131758776	131769186	131791495	131792921	6.5	*C5orf56*
**rs886285**	131758776	131769186	131814628	131819987	5.6	
**rs886285**	131758776	131769186	131819988	131821287	6.3	
**rs886285**	131758776	131769186	131821288	131825016	7.8	*IRF1*
**rs886285**	131758776	131769186	132153133	132153370	5.1	
**rs886285**	131758776	131769186	132208318	132210410	5.3	*LEAP2*
**rs886285**	131758776	131769186	132219272	132219773	5.0	
**rs886285**	131802698	131812303	131758776	131769186	5.3	*AC116366.6, C5orf56,Y_RNA*
**rs886285**	131825017	131826955	131758776	131769186	7.4	*IRF1*

Locus: rs210143 chr6 (*BAK1*)

SNP	SNP bin start	SNP bin end	Promoter bin start	Promoter bin end	Contact score	Gene TSS
**rs210142, rs210143**	33542615	33551201	33381238	33386615	5.6	*CUTA,PHF1*
**rs210142, rs210143**	33542615	33551201	33386616	33392274	5.2	*SYNGAP1*

**rs210142, rs210143**	33542615	33551201	33255433	33256040	5.5	*WDR46*
**rs210142, rs210143**	33542615	33551201	33358373	33363613	6.2	*KIFC1*
**rs210142, rs210143**	33542615	33551201	33363614	33364413	6.0	
**rs210142, rs210143**	33542615	33551201	33392275	33392971	9.3	
**rs210142, rs210143**	33542615	33551201	33392972	33396741	7.9	
**rs210142, rs210143**	33542615	33551201	33396742	33402944	7.3	*SYNGAP1*
**rs210142, rs210143**	33542615	33551201	33402945	33403900	6.3	
**rs210142, rs210143**	33542615	33551201	33404397	33408750	7.5	*MIR5004, SYNGAP1*
**rs210142, rs210143**	33542615	33551201	33408751	33418097	5.5	*SYNGAP1*
**rs210142, rs210143**	33542615	33551201	33423659	33424754	5.8	
**rs210142, rs210143**	33542615	33551201	33655559	33666990	5.6	*MIR3934,SBP1, UQCC2*
**rs210142, rs210143**	33542615	33551201	33666991	33679108	5.5	

Supplementary Table 10: eQTL analysis of lead risk SNPs.

Locus	rsID	Blood genes	Blood P-values	MuTHER genes	MuTHER P-values	CAGE genes	CAGE P-values
6p21.31 (***BAK1***)	**rs210143**	*BAK1*<br>*ITPR3*<br>*MNF1*<br>*PHF1,CUTA*	3.3E-310<br>3.1E-21<br>10.0E-6<br>1.48569E-5	*na*	na	*HLA-DOA*<br>*BAK1*<br>*ITPR3*	2E-13<br>1.3E-36<br>7.9E-08
	rs210142	*BAK1*<br>*ITPR3*<br>*MNF1*<br>*PHF1,CUTA*	3.3E-310<br>3.1E-21<br>1.1E-5<br>1.3E-5	*HLA-DOA*<br>*FLJ43752*<br>*HLA-DPB1*<br>*HLA-DOB*<br>*HLA-DPB2*<br>*TAP1*	2.3E-4<br>1.1E-2<br>5.7E-5<br>5.9E-3<br>1.7E-2<br>2.2E-2	*HLA-DOA*<br>*BAK1*<br>*ITPR3*	1.2E-13<br>7.2E-38<br>1E-08
	rs210134	*BAK1*<br>*ITPR3*<br>*DAXX*<br>*PHF1,CUTA*	3.7E-289<br>2.2E-16<br>4.4E-11<br>6.7E-5	*HLA-DOA*<br>*FLJ43752*<br>*HLA-DPB1*<br>*HLA-DOB*<br>*HLA-DPB2*	3.8E-04<br>5.0E-2<br>4.1E-05<br>1.2E-2<br>1.2E-2	*HLA-DOA*<br>*BAK1*	7.2E-14<br>1.4E-33
5q31.1 (***C5orf56***)	**rs886285**	*SLC22A5*<br>*C5orf56*<br>*Y_RNA*<br>*SLC22A4*<br>*C5orf56*	1.0E-47<br>4.5E-13<br>1.7E-12<br>4.7E-10<br>8.9E-5	*HSPA4*<br>*CSF2*<br>*RAPGEF6*<br>*SLC22A5*<br>*RAD50*	3.8E-2<br>3.1 -2<br>1.6E-2<br>9.1E-06<br>1.3E-05	na	na
	rs2522055	*SLC22A5*<br>*C5orf56*<br>*Y_RNA*<br>*SLC22A4*<br>*C5orf56*	2.7E-46<br>5.3E-14<br>7.5E-14<br>5.6E-10<br>8.6E-5	*na*	na	na	na
	rs2706387	*SLC22A5*<br>*C5orf56*<br>*SLC22A4*	5.1E-50<br>3.9E-12<br>4.7E-12	*HSPA4*<br>*FSTL4*<br>*RAPGEF6*	2.3E-2<br>2.3E-2<br>1.6E-2	na	na

		Y_RNA	2.5E-11	*SLC22A5*<br>*SHROOM1*<br>*RAD50*	6.8E-06<br>3.5E-2<br>5.5E-06		
5q31.1 (***C5orf56***)	rs6865438	*SLC22A5*<br>*SLC22A4*<br>*C5orf56*<br>*Y_RNA*	2.2E-50<br>4.1E-12<br>4.8E-12<br>2.72712E-11	na	na	na	na
	rs7703230	*SLC22A5*<br>*C5orf56*<br>*SLC22A4*<br>*Y_RNA*	2.1E-51<br>3.7E-12<br>4.1E-12<br>3.3E-11	*HSPA4*<br>*FSTL4*<br>*RAPGEF6*<br>*SLC22A5*<br>*SHROOM1*<br>*RAD50*	2.2E-2<br>4.0E-2<br>1.8E-2<br>3.1E-06<br>3.7E-2<br>5.5-06	na	na
	rs4475253	*SLC22A5*<br>*C5orf56*<br>*Y_RNA*<br>*SLC22A4*	8.0E-45<br>2.1E-14<br>3.0E-13<br>3.5E-10	*HSPA4*<br>*CSF2*<br>*FSTL4*<br>*RAPGEF6*<br>*SLC22A5*<br>*RAD50*	3.6E-2<br>3.4E-2<br>4.5E-2<br>1.6E-2<br>1.0E-05<br>1.3E-05	na	na
	rs726923	*SLC22A5*<br>*SLC22A4*<br>*C5orf56*<br>*Y_RNA*	1.9E-51<br>3.4E-12<br>3.9E-12<br>2.9E-11	*RAPGEF6*<br>*SLC22A5*<br>*LEAP2*<br>*SHROOM1*<br>*RAD50*	5.8E-3<br>1.5E-05<br>4.4E-2<br>3.1E-2<br>6.6E-07	na	na
	rs12659708	*SLC22A5*<br>*C5orf56*<br>*SLC22A4*<br>*Y_RNA*	6.4E-50<br>3.9E-12<br>5.0E-12<br>2.5E-11	na	na	na	na
	rs2522043	*SLC22A5*<br>*SLC22A4*<br>*C5orf56*<br>*Y_RNA*	9.5E-51<br>3.8E-12<br>7.3E-12<br>3.2E-11	na	na	na	na
	rs12655512	*SLC22A5*	6.4E-50	*HSPA4*	2.3E-2	na	na

		C5orf56<br>*SLC22A4*<br>*Y_RNA*	3.9E-12<br>5.0E-12<br>2.47907E-11	*FSTL4*<br>*RAPGEF6*<br>*SLC22A5*<br>*SHROOM1*<br>*RAD50*	2.3E-2<br>1.6E-2<br>6.7E-06<br>3.5E-2<br>5.5E-06		
5q31.1 (***C5orf56***)	rs2522044	*SLC22A5*<br>*SLC22A4*<br>*C5orf56*<br>*Y_RNA*	8.8E-51<br>3.4E-12<br>7.3E-12<br>3.2E-11	*HSPA4*<br>*FSTL4*<br>*RAPGEF6*<br>*SLC22A5*<br>*SHROOM1*<br>*RAD50*	2.3E-2<br>2.3E-2<br>1.6E-2<br>6.6E-06<br>3.5E-2<br>5.5E-06	na	na
	rs4504381	*SLC22A5*<br>*C5orf56*<br>*SLC22A4*<br>*Y_RNA*	2.2E-50<br>2.0E-12<br>3.4E-12<br>2.1216E-11	*HSPA4*<br>*FSTL4*<br>*RAPGEF6*<br>*SLC22A5*<br>*SHROOM1*<br>*RAD50*	3.1E-2<br>2.9E-2<br>1.4E-2<br>4.8E-06<br>3.2E-2<br>4.6E-06	na	na
	rs10059611	*SLC22A5*<br>*SLC22A4*<br>*C5orf56*<br>*Y_RNA*	4.9E-51<br>3.0E-12<br>1.1E-11<br>3.3E-11	*HSPA4*<br>*FSTL4*<br>*RAPGEF6*<br>*SLC22A5*<br>*SHROOM1*<br>*RAD50*	2.5E-2<br>2.5.E-2<br>1.5E-2<br>5.790E-06<br>0.0339<br>5.134E-06	na	na
	rs7713818	*SLC22A5*<br>*C5orf56*<br>*SLC22A4*<br>*Y_RNA*	5.1E-50<br>3.9E-12<br>4.6E-12<br>2.5E-11	*HSPA4*<br>*FSTL4*<br>*RAPGEF6*<br>*SLC22A5*<br>*SHROOM1*<br>*RAD50*	2.3E-2<br>2.3E-2<br>1.6E-2<br>6.6E-06<br>3.5E-2<br>5.5E-06	na	na
	rs6866467	*SLC22A5*<br>*SLC22A4*<br>*C5orf56*<br>*Y_RNA*	1.7E-52<br>3.0E-12<br>1.2E-11<br>2.1E-10	*RAPGEF6*<br>*SLC22A5*<br>*SHROOM1*<br>*RAD50*	1.6E-2<br>3.0E-05<br>5.0E-2<br>2.1E-06	na	na

				P4HA2	1.0E-2		
21q22.3 (***ERG***)	**rs9976326**	na	na	*BRWD1*	2.8E-2	na	na
	rs55681902	na	na	na	na	na	na
17q21.32 (***IGF2BP1***)	**rs10853104**	*UBE2Z*<br>*ATP5G1*	1.9E-53<br>8.0E-15	*DLX4*<br>*UBE2Z*<br>*ATP5G1*	8.2E-3<br>3.5E-05<br>1.8E-07	*ATP5G1*	7.5E-08
9q21.3 (***TLE1***)	**rs76925697**	na	na	na	na	na	na
	rs62579834	na	na	na	na	na	na
	rs62579836	na	na	na	na	na	na
	rs62579838	na	na	na	na	na	na
	rs62579841	na	na	na	na	na	na
	rs868524	na	na	na	na	na	na

Date sources : CAGE[8], MuTHER[9], Blood[10]. Lead SNPs shown in bold.

Supplementary Table 11: Predicted transcription factor disruption

Chromosome 6p21.3 nearest gene *BAK1*

SNP rsID<br>**Ref**|Alt	ChIP-seq<br>Blood Cells	Predicted motif<br>Disruption	PhastCons	GERP
rs210134<br>**A**|G		ETS1,HAND1,SMC3	0	3.6
rs210142<br>**T**|C	BRD4, MYC, TCF3, CTCF	**CEBP, EHF, ELF1, ELF5, ETS, ETV4,** FOS, **GATA2, GTF2I**, UA3, **RAR, SPI1, SPIB**, SPZ1, **STAT[1,3,4,5A,6],** TCF12, TEAD2, WT1	0	1.43
rs210143<br>**T**|C	CEBPB, CTCF	**CEBPA, NFIC,** SMAD3, **TFCP2, TLX1**	0	1.43

Chromosome 5q31.1 nearest genes *IRF1, C5orf56*

SNP rsID<br>**Ref**|Alt	ChIP-seq<br>Haematopoietic cells	Predicted motif<br>Disruption	PhastCons	GERP
rs10059611<br>**G**|T		DBP, **E2F1, ELF1**	0	-1.55
rs12655512<br>**C**|T		FOXL1, HLTF, **SNAI2, ZEB1**	0	1.3
rs12659708<br>**A**|G		ATF3, CACD, CCNT2, CHD2, E2F1, GABPA, IRF1, KLF[4,5,7], MAX, MAZ, SP1, **RAR,** SP[1..3], SREBF2, TBP, TEAD2, **TP53, TP73,** ZBTB33	0	-1.48
rs2522043<br>**T**|A	BRD4, EP300, JMJD1C, MED1, PBX1, PML, RUNX3, STAT4, TCF3, EBF1, IKZF1, RUNX1	**HNF4A,** RAD21, RAR, RHOXF1, **SMC3,** SNAI2, ZEB1	0	0.54
rs2522044<br>**C**|T	EBF1, IKZF1, RUNX1	**ATOH1,** FOXH1, FOXO3, **GATA[1..3,6],** HMGA1, **LMO2, MYB, MYBL2, MYF6, MYOD1, MYOG,** ZEB1, **NEUROD1, PTF1A,** SOX10, SOX13, SOX3, SOX4,	0	-0.34

	SOX6, SRY, **TAL1, TCF12, TCF3, TCF4, TFAP4**		
rs2706387<br>**C**|T	AP3, **ASCL2,** BRCA1, **FIGLA, FOXA1, MAFB,** MYB, MYBL2, **MYF5, MYOD1, MYOG,** ZEB1, **NHLH1, NR3C1, PTF1A,** RFX1, **SIN3A, SMC3, TAF1, TCF12, TCF3, TFAP4, TFCP2, TFCP2L1**	0.006	2.38
rs4475253<br>**A**|G	**ARID5B,** ATF3, **BARX1, BSX, CRX, EN1, EVX1, EVX2, GATA[1..6], GSC2, GCS, GSX2, HMGA1, HMGN3, HOXB2, HOXB3, ISL1,** LMO2, **GATA1,NFE2L1, NFE2L1::MAFG, NKX6-2, PDX1, PITX1, POU2F1,** SIX[1,2,3,4,6]	0.019	2.02
rs4504381<br>**T**|G	**DBP, EN1, FUBP1, HDAC2, HES1, HOXD10, NKX2-1, NKX3-2,** TFAP2A	0	1.82
rs6865438<br>**T**|C	ELF1, FOS, IKZF2, MECP2, UA9, **NANOG, USF2,** ZIC1, **ZNF143,** ZNF350	0	-0.98
rs6866467<br>**A**|G	IKZF2, TAL1, **NKX2-5,** ZNF263	0	0.04
rs726923<br>**C**|A	**CEBPA, CEBPB, CEBP, ELF5, ETS1, ETS, FLI1,** FOXL1, TEAD1, NFE2L1, POU5F1, **STAT3, STAT, TEAD[1..4]**	0	-0.59
rs7703230<br>**G**|T	ALX1, ALX3, ARID3A, BARHL1, BARHL2, BARX1, BARX2, BSX, CEBPD, DLX[1..6], EMX1, EMX2, EN1, EN2, ESX1, EVX1, EVX2 **FOXC1,** GBX1, GBX2, GSX1, GSX2, HESX1, HMGA1, HMX[1..3], HOXA[1,2,5,10,11], HOXB[2,3,5,8], HOXC10, HOXC11, HOXD[3,4,8,9], ISL1, ISL2, ISX, LBX2, LHX[3,4,8,9], LMX1A, LMX1B, MEOX2, MIXL1, MNX1, MSX1, MSX2, NANOG, NKX1-2, **NKX2-1,** NKX2-5, NKX6-1, NKX6-2, NOBOX, PAX4, PAX7, PDX1, PRRX1, PRRX2, RAX2, RAX, SHOX2, SHOX, **SIX1, TEAD1,**	0.288	2.82

		**TP53,** UNCX, VAX1, VENTXl, VSX1		
rs7713818<br>**C**|T	CTCF	**ETS1,** HLTF, ZNF263, RXRA, STAT4, STAT6, **WT1, ZNF263**	0	-0.04
rs886285<br>**T**|C		ISL1, **LMX1B, MEOX1, MNX1, NKX6-2, NKX6-3, POU4F3, POU6F1, POU6F2,** VENTX	0.079	2.4
rs2522055<br>**A**|C	BCL6, BRD4, IKZF1, TCF3		0	0.53

Chromosome 21q22.2 nearest gene *ERG*

SNP rsID<br>**Ref**|Alt	ChIP-seq<br>Haematopoietic cells	Predicted motif<br>Disruption	PhastCons	GERP
rs55681902<br>**T**|C		**ALX1, ALX3, BSX, CEBP,** CRX, CTCF, DLX1, DLX2, DLX3, DLX4, **DLX5, DLX6, E2F1, EMX2, EN1, EN2, ESX1,** GATA[1..6], **GBX1, GBX2,** GFI1, **HESX1, HMGA1, HOXA5, HOXD4, ISX, LBX2, LHX2, LMX1B, MSX1, MSX2, NKX6-2, NOBOX, NOTO,** OTX1, OTX2, OTX, **PAX2, PAX4,** PITX1, **PRRX1, PRRX2,** RAD21, **RAX2, RAX, SHOX2, SHOX, UNCX, VSX1, VSX2**	0	0.67
rs9976326<br>**A**|T	EBF1, EP300, IKZF1, LEO1, MED1, NR3C1, PBX1, RUNX1, TCF3	AFP, ARID3A, **BARHL1, BARHL2,** BARX1, BARX2, BSX, **CDX2, CEBPB, CEBP,** CRX, DLX3, EN2, EVX1, EVX2, **FOXC1,** GSX1, GSX2, **HMGA1,** HNF1A, HOXA11, HOXA2, HOXB2, HOXB3, HOXC10, HOXD3, HOXD9, ISL1, **ISL2,** LHX9, MYB, NANOG, NKX2-5, NKX6-1, **NKX6-2,** NOTO, **PAX2,** PDX1, POU2F1, RUNX1, **STAT1, TLX2,** VENTX	0	-2.5

Chromosome 17q21.3 nearest gene *IGF2BP1*

SNP rsID<br>**Ref**|Alt	ChIP-seq<br>Haematopoietic cells	Predicted motif<br>Disruption	PhastCons	GERP
rs10853104<br>**C**|T	BCL6, BRD4, CTCF, CTCFL, ERG, FLI1, GATA1, KLF1, KMT2A, NFE2, RAD21, SMAD1, STAG1, TCF3, TRIM28	CTCF**, E2F1, ELF1, ESRRA,** HIC1, MYF, NF1, SMAD3, **TCF12, TEAD2,** TFCP2, **ZBTB7A, (NEF2)**	0	-2.56

Chromosome 9q21.3 nearest gene *TLE1*

SNP rsID<br>**Ref**|Alt	ChIP-seq<br>Haematopoietic cells	Predicted motif<br>Disruption	PhastCons	GERP
rs62579834<br>**C**|A		AR, EBOX, ENO1, HNF4A, **ITGB2,** LMO2, MITF, MYC::MAX, MYF6, NR3C1, OLIG2, PGR ,TFE3, TFEC, TFE, USF2, USF, **YY1**	0	-0.25
rs62579836<br>**G**|C		**IRF4,** KLF6, **MAZ,** MEIS3, **POLR3A,** SMAD	0.409	-0.513
rs62579838<br>**T**|G		**ALX[1,3], ARID3A, BARHL1, BARX[1,2], BSX,** CEBP, **CRX, DLX[1..6], EMX[1,2], EMX2, EN[1,2] ,ESX1, EVX[1,2],** FOXA[1,2], FOXB1, FOXC1, FOXD[1,3], FOXF2, FOXG1, FOXI1, **FOXP3, GBX[1,2], GSX[1,2],** HDAC2, **HESX1,** HLTF, **HMGA1, HOXA[1,2], HOXB[2,3,5], HOXC10, HOXD[3,4,8,9], ISX, LBX2,** LEF1, **LHX[2,4,9] ,LMX1[A,B], MIXL1, MNX1, MSX[1,2], NKX2-5, NKX6-[1,2], NOBOX, NOTO, PAX[4,7], PAX7, PBX1, PDX1, POU3F2, PRRX[1,2], RAX2, RAX, SHOX,** SOX[2,4,10,11,15], TCF7, **TLX2, UNCX, VSX[1,2]**	0	-3.39
rs62579841<br>**T**|C		FOXN1, GMEB2, **HMGA1,** MYB, **PAX7, POU1F1, TEAD1,** ZEB1	0	2.27
rs76925697	BRD4, CREBBP, EP300,	BARHL1, BARHL2, CDX2, CPEB1,	0.278	0.00

**A**|T	ERG, FLI1, GATA2, GATA3, IKZF1, KDM6A, MED1 ,MYB, NFE2, NOTCH1, PBX1, RUNX1 ,SKI, SMAD1, SPI1, TAL1, TCF12, TCF3, TCF7L2, TLX1, TRIM28	FOXC1, **HAND1,** HOXA11, HOXD13 ,**IRF[1..3,7,9], STAT[,1,4,5A,6], UBP1, ZNF384**		
rs868524<br>**G**|A		ATF4, CEBPA, CEBPB ,CEBPD, CEBP, EP300, **MEIS3, CEBPB, SMAD, SOX13, SOX17, SOX30, SOX6, SOX9, SRY,** TFAP2A	0	-1.9

Description: transcription factor binding sites downloaded from ChIP-Atlas (http://chip-atlas.org/) filtering for cell lines with a haematopoietic lineage and a MACS peak Q value of greater than 100. Overlapping binding sites from the same ChIP target were merged. Predicted motif disruption was performed using MotifbreakR[11], filtering for targets with a predicted strong effect and targets with a best matching allele score of >0.9 (max=1). Targets with ChIP peaks and predicted disrupted binding at a SNP are highlighted in brackets. NEF2 binding is predicted disrupted by rs10853104 with low effect but was included, in brackets, due to matched ChIP binding site. Proteins with a predicted greater affinity for the reference allele of a SNP are shown in bold. When multiple proteins from the same family are predicted to have their binding disrupted these have been abbreviated, for example STAT[,1..3,5A,6] represents STAT, STAT1, STAT2, STAT5A and STAT6. PhastCons and GERP evolutionary conservation scores were extracted from UCSC datasets 'phastConsElements46way.txt.gz' 'allHg19RS_BW.txt.gz' respectively, all data based on Hg19 build.

Supplementary Table 12: Results from the SMR eQTL and mQTL analysis of the combined summary GWAS datasets. P_{SMR}shows the significant summary *P* value from the analysis after filtering each dataset specific thresholds. P_{HEIDI} shows the heterogeneity associated with each probe-SNP analysis (value <0.05 indicates significant heterogeneity). Loci that pass the P_{SMR} threshold for each gene expression dataset combined with a P_{HEIDI} > 0.05 are considered as regions in which risk alleles contribute to ALL risk by influencing gene expression in-cis. Columns show Tissue: The dataset used in the analysis; CHR: chromosome; Probe ID; Gene: gene symbol; Probe bp: probe position; SNP: rsID; SNP bp: SNP position; A1: effect allele; A2: alternate allele; Freq: allele frequency of A1; b_{GWAS}: Beta from GWAS; SE from GWAS; *P*-value from GWAS; b_{eQTL}: effect size from eQTL; SE from eQTL; $P_{eQTL:}$ *P*-value from eQTL dataset; b_{XY}: effect size from SMR analysis; SE from SMR analysis; *P*-value from SMR analysis; P_{HEIDI}: *P*-value from HEIDI (Heterogeneity in Dependent Instruments); number of SNPs used in HEIDI test

**Tissue**	**CHR**	**Probe ID**	**Gene**	**Probe bp**	**SNP**	**SNP bp**	**A1**	**A2**	**Freq (A1)**
Blood eQTL; Westra *et al*	10	ILMN_2152465	*PIP4K2A*	22826096	rs10764339	22867210	T	C	0.38
Blood eQTL; GTEx	6	ENSG00000030110.8	*BAK1*	33544174	rs210143	33546930	C	T	0.71
Lymphoctye eQTL; GTEx	6	ENSG00000030110.8	*BAK1*	33544174	rs210134	33540209	G	A	0.69
mQTL (Aberdeen); Hannon *et al*	10	cg13344587	*ARID5B*	63723919	rs7896246	63724390	A	G	0.34
mQTL (Aberdeen); Hannon *et al*	7	cg01139861	*IKZF1*	50343298	rs11761922	50344132	C	G	0.31
mQTL (Aberdeen); Hannon *et al*	7	cg14216940	*IKZF1*	50343131	rs11761922	50344132	C	G	0.31
mQTL (Aberdeen); Hannon *et al*	21	cg27471246	*ERG*	39776434	rs9976326	39776485	T	A	0.24
mQTL (UCL); Hannon *et al*	7	cg14216940	*IKZF1*	50343131	rs6583437	50350267	A	G	0.38
mQTL (UCL); Hannon *et al*	10	cg20641026	*PIP4K2A*	22906638	rs1750775	22938848	C	T	0.32
mQTL (UCL); Hannon *et al*	12	cg18954900	*ELK3*	96604497	rs12828252	96612094	G	A	0.30
mQTL (UCL); Hannon *et al*	21	cg27471246	*ERG*	39776434	rs2836364	39767874	T	C	0.25

b_{GWAS}	SE_{GWAS}	P_{GWAS}	b_{eQTL}	SE_{eQTL}	P_{eQTL}	b_{XY}	*SE*	P_{SMR}	P_{HEIDI}	$nSNP_{HET}$
-0.170	0.027	2.89X10^{-10}	-0.564	0.019	4.69X10^{-202}	0.302	0.049	6.71X10^{-10}	5.56X10^{-02}	19
0.138	0.029	1.74X10^{-06}	-0.624	0.038	2.01X10^{-59}	-0.221	0.048	4.48X10^{-06}	6.32X10^{-01}	19
0.131	0.028	4.17X10^{-06}	-0.765	0.077	3.67X10^{-23}	-0.171	0.041	2.99X10^{-05}	2.61X10^{-01}	19
0.584	0.027	9.13X10^{-106}	-0.040	0.002	2.01X10^{-118}	-14.500	0.913	8.36X10^{-57}	8.98X10^{-01}	13
0.208	0.028	2.14X10^{-13}	0.060	0.002	8.27X10^{-160}	3.489	0.493	1.43X10^{-12}	5.36X10^{-02}	19
0.208	0.028	2.14X10^{-13}	0.049	0.002	1.01X10^{-135}	4.281	0.608	1.95X10^{-12}	7.40X10^{-02}	18
0.171	0.030	1.11X10^{-08}	-0.019	0.002	2.21X10^{-26}	-8.889	1.767	4.86X10^{-07}	5.31X10^{-02}	10
0.178	0.027	3.08X10^{-11}	0.037	0.003	1.36X10^{-46}	4.760	0.790	1.67X10^{-09}	2.76X10^{-01}	2
-0.168	0.028	2.02X10^{-09}	0.026	0.003	3.72X10^{-15}	-6.428	1.348	1.86X10^{-06}	7.66X10^{-02}	7
0.143	0.028	3.53X10^{-07}	0.027	0.003	1.93X10^{-20}	5.374	1.204	8.08X10^{-06}	3.26X10^{-01}	5
0.153	0.029	1.56X10^{-07}	-0.019	0.002	6.61X10^{-16}	-8.127	1.848	1.09X10^{-05}	2.04X10^{-01}	6

Supplementary Table 13: Details of the datasets used for the SMR analysis detailing P_{smr} and P_{HEIDI} thresholds.

**Study**	**Number of SMR tests**	**P_{SMR} threshold**	**Genes passing P_{SMR} threshold**	**Genes passing P_{HEIDI} (>0.05) threshold**
Blood eQTL; Westra *et al*	5850	$< 8.5 \times 10^{-6}$	5	1
Blood eQTL; GTEx	4133	$< 1.2 \times 10^{-5}$	3	1
EBV transformed Lymphoctye eQTL; GTEx	1388	$< 3.6 \times 10^{-5}$	2	1
mQTL (Aberdeen)	41098	$< 1.2 \times 10^{-6}$	10	0
mQTL (UCL)	29926	$< 1.6 \times 10^{-6}$	6	1

Supplementary Table 14: Heritability of childhood acute lymphoblastic leukaemia explained by genome-wide SNPs estimated using Linkage Disequilibrium Adjusted Kinship (LDAK) method.

Total SNPs used	Heritability	Standard deviation
1,553,634	21%	0.065

Supplementary Table 15: Individual variance in risk associated with the most significant SNPs from each risk loci associated with childhood ALL. CHR: Chromosome; RAF: risk allele frequency. *New loci discovered in current meta-analyses. OR: Odds ratio; CI: confidence intervals. OR and CI are derived from current meta-analysis. CHR: chromosome; BP: base pair; % of total variance in risk to childhood ALL was tested under the following Standardised incidence ratios (SIR) of 3.2 (95% CI: 1.5-5.9) as per Kharazmi *et al*.

CHR	SNP (Subtype)	Locus (gene)	Position (BP)	Risk Allele	RAF	OR (95% CI)	P-value	% of total variance in risk explained
2	rs17481869(*ETV6-RUNX1*)	2q22.3	146124454	A	0.08	1.74 (1.45-2.09)	$2.37x10^{-09}$	2.54
5	*rs886285 (High-hyperdiploidy)	5q31.1 (*C5orf56*)	131765206	T	0.34	1.29 (1.18-1.41)	$1.56x10^{-08}$	1.61
6	*rs210143 (High-hyperdiploidy)	6p21.31 (*BAK1*)	33546930	C	0.73	1.30 (1.19-1.43)	$2.21x10^{-08}$	1.55
7	rs17133805	7p12.2 (*IKZF1*)	50477514	G	0.32	1.65 (1.56-1.74)	$5.28x10^{-71}$	6.10
8	rs75777619	8q24.21	130185176	G	0.12	1.26 (1.17-1.36)	$2.30x10^{-09}$	0.62
9	rs113650570	9p21.3 (*CDKN2A*)	21976402	A	0.02	2.32 (2.03-2.65)	$8.06x10^{-35}$	1.82
9	*rs76925697	9q21.31	83747371	A	0.96	1.52 (1.31-1.76)	$2.11x10^{-08}$	0.75
10	rs10821936	10q21.2 (*ARID5B*)	63723577	C	0.33	1.80 (1.71-1.89)	$1.19x10^{-106}$	8.50
10	rs3824662	10p14 (*GATA3*)	8104208	A	0.19	1.29 (1.21-1.38)	$3.57x10^{-14}$	1.11
10	rs2296624	10p12.2 (*PIP4K2A*)	22856946	C	0.67	1.25 (1.18-1.32)	$2.79x10^{-15}$	1.19
10	rs12779301	10q26.13 (*LHPP*)	126292655	C	0.66	1.22 (1.15-1.29)	$5.72x10^{-13}$	0.98
14	rs2239630	14q11.2 (*CEBPE*)	23589349	A	0.45	1.28 (1.22-1.35)	$1.72x10^{-21}$	1.72
17	*rs10853104 (*ETV6-RUNX1*)	17q21.32 (*IGF2BP1*)	47092076	T	0.47	1.33 (1.21-1.47)	$1.82x10^{-08}$	2.31
21	rs9976326	21q22.2 (*ERG*)	39776485	T	0.25	1.19 (1.12-1.26)	$1.11x10^{-08}$	0.63
						**Overall %**		**31.43%**

Supplementary Table 16: Conditional and joint analysis using summary statistics under a stepwise model (GCTA cojo analysis). CHR: chromosome; bp: base pair; refA: allele tested; freq: frequency; b: Beta; se: standard error; bj: joint analysis beta; bj_se: joint analysis standard error; LD correlation between SNP i and SNP i + 1 from SNPs on the list.

CHR	SNP	bp	refA	freq	b	se	Meta *P*-value	n	freq_geno	bJ	bJ_se	COJO *P*-value	LD
7	rs17133805	50477514	G	0.34	0.50	0.03	5.28E-71	20844.3	0.28	0.50	0.03	5.71E-70	0
8	rs75777619	130185176	G	0.14	0.23	0.04	2.30E-09	20776.8	0.12	0.23	0.04	2.37E-09	0
9	rs113650570	21976402	A	0.05	0.84	0.07	8.06E-35	18497.2	0.03	0.63	0.07	8.27E-18	-0.15
9	rs10811644	22025067	T	0.44	-0.19	0.03	8.66E-13	21993.4	0.46	-2.23	0.07	4.20E-200	0.94
9	rs10757266	22049555	T	0.46	0.11	0.03	2.73E-05	22181.1	0.48	2.79	0.08	2.44E-273	0.66
9	rs944800	22050898	G	0.66	-0.14	0.03	4.12E-07	21949	0.68	-0.71	0.04	2.30E-63	0.19
9	rs17694555	22051295	G	0.11	0.40	0.04	9.52E-20	20385.9	0.08	0.78	0.06	3.31E-45	0
9	rs76925697	83747371	T	0.03	-0.42	0.07	2.11E-08	24233.5	0.04	-0.42	0.07	2.16E-08	0
10	rs10821936	63723577	T	0.59	-0.59	0.03	1.19E-106	21186.2	0.66	-0.59	0.03	2.48E-104	0
10	rs3824662	8104208	A	0.21	0.25	0.03	3.57E-14	20222.8	0.17	0.25	0.03	3.87E-14	0
10	rs4335448	22359075	G	0.76	0.20	0.03	6.05E-11	23252.5	0.75	0.18	0.03	1.42E-09	0
10	rs74229974	22476221	T	0.20	-0.22	0.03	6.37E-12	23482.8	0.23	-0.18	0.03	9.17E-09	0.16
10	rs2296624	22856946	T	0.31	-0.22	0.03	2.79E-15	22650.7	0.34	-0.18	0.03	1.34E-10	0
10	rs12779301	126292655	C	0.66	0.20	0.03	5.72E-13	22203.7	0.63	0.20	0.03	6.08E-13	0
21	rs9976326	39776485	T	0.27	0.17	0.03	1.11E-08	21262.2	0.24	0.17	0.03	6.55E-09	-0.02
21	rs9979478	39807498	T	0.93	0.27	0.05	1.90E-08	23146.2	0.91	0.28	0.05	1.11E-08	0

References

1. Tian, L. *et al.* Long-read sequencing unveils IGH-DUX4 translocation into the silenced IGH allele in B-cell acute lymphoblastic leukemia. *Nat Commun* **10**, 2789 (2019).
2. Rao, S.S. *et al.* A 3D map of the human genome at kilobase resolution reveals principles of chromatin looping. *Cell* **159**, 1665-80 (2014).
3. Javierre, B.M. *et al.* Lineage-specific genome architecture links enhancers and non-coding disease variants to target gene promoters. *Cell* **167**, 1369-1384. e19 (2016).
4. Cairns, J. *et al.* CHiCAGO: robust detection of DNA looping interactions in Capture Hi-C data. *Genome Biol* **17**, 127 (2016).
5. Qian, M. *et al.* Whole-transcriptome sequencing identifies a distinct subtype of acute lymphoblastic leukemia with predominant genomic abnormalities of EP300 and CREBBP. *Genome Res* **27**, 185-195 (2017).
6. Alexander, D.H., Novembre, J. & Lange, K. Fast model-based estimation of ancestry in unrelated individuals. *Genome Res* **19**, 1655-64 (2009).
7. Perez-Andreu, V. *et al.* Inherited GATA3 variants are associated with Ph-like childhood acute lymphoblastic leukemia and risk of relapse. *Nat Genet* **45**, 1494-8 (2013).
8. Lloyd-Jones, L.R. *et al.* The Genetic Architecture of Gene Expression in Peripheral Blood. *Am J Hum Genet* **100**, 371 (2017).
9. Grundberg, E. *et al.* Mapping cis- and trans-regulatory effects across multiple tissues in twins. *Nat Genet* **44**, 1084-9 (2012).
10. Westra, H.J. *et al.* Systematic identification of trans eQTLs as putative drivers of known disease associations. *Nat Genet* **45**, 1238-1243 (2013).
11. Coetzee, S.G., Coetzee, G.A. & Hazelett, D.J. motifbreakR: an R/Bioconductor package for predicting variant effects at transcription factor binding sites. *Bioinformatics* **31**, 3847-9 (2015).
